# Transcription factor NF-kappa B represses ANT1 transcription and leads to mitochondrial dysfunctions

**DOI:** 10.1038/srep44708

**Published:** 2017-03-20

**Authors:** Chen Zhang, Hui Jiang, Pin Wang, Heng Liu, Xiulian Sun

**Affiliations:** 1 Department of Neurology, Qilu Hospital of Shandong University, No. 107 West Wenhua Road, Jinan, 250012, Shandong Province, China; 2 Department of Pediatrics, 2nd Hospital of Shandong University, No. 44 West Wenhua Road, Jinan, 250011, Shandong Province, China; 3 Otolaryngology Key, Lab of Ministry of Health, No. 44 West Wenhua Road, Jinan, China; 4 Brain Research Institute, Qilu Hospital of Shandong University, No.107 West Wenhua Road, Jinan, 250012, Shandong Province, China

## Abstract

Mitochondria are intracellular organelles involved in cell survival and death, and dysfunctions of mitochondria are related to neurodegenerative diseases. As the most abundant protein in the mitochondrial inner membrane, adenine nucleotide translocator 1 (ANT1) plays a critical role in mitochondrial function, including the exchange of adenosine triphosphate/adenosine diphosphate (ATP/ADP) in mitochondria, basal proton leak and mitochondrial permeability transition pore (mPTP). Here, we show that *ANT1* transcription is regulated by transcription factor NF-kappa B (NF-κB). NF-κB is bound to two NF-κB responsive elements (NREs) located at +1 to +20 bp and +41 to +61 bp in the *ANT1* promoter. An NF-κB signalling stimulator, tumour necrosis factor alpha (TNFα), suppresses *ANT1* mRNA and protein expression. Activation of NF-κB by TNFα impairs ATP/ADP exchange and decreases ATP production in mitochondria. Activation of NF-κB by TNFα decreases calcium induced mPTP opening, elevates mitochondrial potential and increases reactive oxygen species (ROS) production in both T98G human glioblastoma cells and rat cortical neurons. These results demonstrate that NF-κB signalling may repress *ANT1* gene transcription and impair mitochondrial functions.

Mitochondria are responsible for supplying cytoplasmic adenosine triphosphate (ATP) to support cellular activities and are the main intracellular source of ROS^1^. Increasing evidence implicated the accompanied role of mitochondrial dysfunction in age-related neurodegenerative disorders, including Alzheimer’s disease (AD), Parkinson’s disease (PD) and Huntington’s disease (HD)^2^,^3^,^4^. The presence of neuroinflammation and oxidative stress is also a common feature in neurodegenerative diseases. Thus, the mechanisms underlying mitochondrial dysfunctions and inflammation signalling require clarification.

Adenine nucleotide translocator 1 (ANT1), located in the inner mitochondrial membrane, accounts for up to 10 percent of total mitochondrial protein contents^5^. Encoded by different nuclear genes, four ANT1 isoforms (hANT1, hANT2, hANT3, hANT4) have been reported in humans with different tissue distribution^6^,^7^. While in mice, only three ANT isoforms, designated mANT1, mANT2, and mANT4, have been described^8^,^9^. Transcriptional coactivator PGC-1a, acting via different transcription factors depending on different cell types, controls both the human and mouse ANT1 isoform genes though different mechanisms^10^. As a member of the mitochondrial carrier family (MCF) of proteins, ANT1 plays a key role in catalysing the exchange of cytosolic adenosine diphosphate (ADP) to intramitochondrial ATP for cellular energy supply^11^,^12^. Recent study has shown that suppression of ANT contributes to low cytosolic ATP/ADP, activation of the import of glycolytic ATP into mitochondria of cancer cells^13^. As the driving force in phosphorylation of ADP to ATP, the normal level of mitochondrial membrane potential (Δψm) is also maintained by ANT1-dependent ATP/ADP exchange^14^. Irrelevant to its function in ATP/ADP translocase, ANT1 also plays a significant regulatory role in basal uncoupling or proton leak^15^,^16^,^17^. ANT1 accounts for one- to two-thirds of basal proton conductance through an unknown mechanism, and ANT1-deficient mice exhibit a 50 percent decrease in proton conductance in skeletal muscle^18^. Along with the voltage-dependent anion channel (VDAC) in the outer membrane and cyclophilin D (CyPD) in the matrix, ANT1 is another component of the mitochondrial permeability transition pore (mPTP) complex, a non-specific pore permeable to any molecule smaller than 1.5 kDa, which opens in the inner mitochondrial membrane under conditions of elevated matrix Ca^2+^ 19,20.

Known as a family of transcription factors, nuclear factor kappa B (NF-κB) is a critical regulator of genes involved in immuno-inflammatory responses, tumorigenesis and apoptosis^21^. Mammalian NF-κB has five constituents: RelA/p65, RelB, c-Rel, NF-κB1 (p50) and NF-κB2 (p52)^22^. Inactivated NF-κB is sequestered in cytoplasm and bounded to the IκB family of inhibitor proteins, including IκBα, IκBβ, IκBγ and IκBε^23^. Stimulated by a variety of inducers, such as tumour necrosis factor α (TNFα) and lipopolysaccharide (LPS), the activated NF-κB sub-units translocate into the nucleus to bind with target genes and regulate their transcriptions, leaving the cytoplasmic partner IκBs phosphorylated and degraded^24^,^25^. Mitochondria are important targets of pro-inflammatory cytokines, and interrelated factors may contribute to the mitochondrial dysfunction associated with inflammation. IL-6, a pro-inflammatory cytokine released in activated glia, has been shown to stimulate ROS accumulation in the brain, contributing to other ROS production mechanisms in inflammation^26^. The inflammatory cytokine TNFα has been shown to induce deterioration of mitochondrial function through suppression of mitochondrial complexes I and IV and pyruvate dehydrogenase activities^27^. It has been shown that LPS-induced inflammation promotes strong microglial activation and induces mitochondrial dysfunction, both *in vitro* and *in vivo*^28^. NF-κB represses mitochondrial gene expression, including cytochrome B and cytochrome C oxidase mRNA levels and NF-κB is found to be located in mitochondria^29^,^30^. Previous studies also showed ANT1 expression was reduced following TNFα or H_2_O_2_ treatment^31^,^32^.

ANT1 plays critical roles in mitochondrial functions; however, its molecular transcription is unknown. It would be interesting to investigate the internal relationship between transcriptional factor NF-κB and *ANT1* gene and potential roles of NF-κB in mitochondrial functions related to ANT1. Our studies here elucidated the mechanism of *ANT1* gene transcription. We found NF-κB repressed *ANT1* gene transcription by binding to two NREs in *ANT1* promoter. Activation of NF-κB by TNFα impaired the ATP/ADP exchange, mPTP opening and ROS production. The study provides a molecular link between inflammation and mitochondrial functions.

## Results

### NF-κB represses ANT1 gene transcription and protein expression

**Figure 1:**
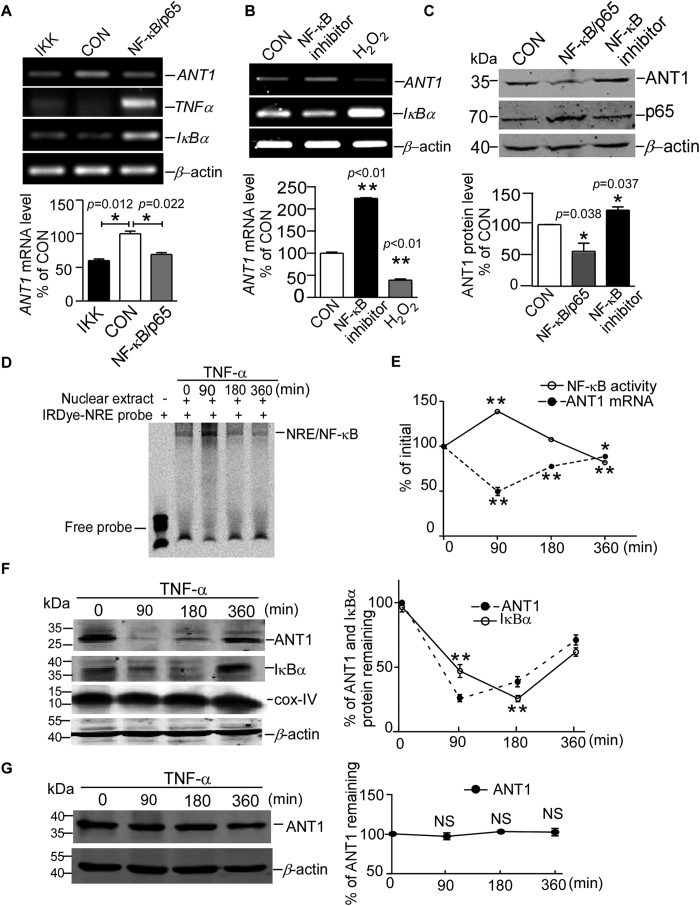
NF-κB represses ANT1 gene transcription and protein expression. (**A**) RT-PCR shows the ANT1, TNFα, and IκBα mRNA expression in HEK 293 cells after 24 hours transfection with IKKβ and NF-κB/p65. The histogram depicts the mean ratios of ANT1 mRNA to β-actin ± SEM (n = 3); *p < 0.05, Student’s t test. (**B**) ANT1 and IκBα mRNA expression in HEK293 were examined in the presence of NF-κB activity inhibitor JSH-23 (15 μM) and the NF-κB activator H_2_O_2_ (50 μM Values represent means ± SEM (n = 3); **p < 0.01, Student’s t test. (**C**) The endogenous ANT1 protein in HEK293 was affected by NF-κB/p65 transfection and NF-κB inhibitor JSH-23 (15 μM). Cropped blots were displayed. Anti-ANT1 (ab110322, Abcam) and anti-NF-κB/p65 (#8242, CST) antibodies were used in WB. β-actin was used as loading controls. Values represent means ± SEM (n = 3); *p < 0.05, Student’s t test. (**D**) The IRDye 800-labelled consensus NRE oligo was used as probes in EMSA. Nuclear extracts in EMSA were extracted from T98G cells treated with TNFα (10 ng/ml) for 0, 90, 180 and 360 minutes. NRE, NF-κB responsive elements (**E**) ANT1 mRNA levels and NF-κB activities in TNFα (10 ng/ml) induced T98G cells for 0, 90, 180 and 360 minutes were depicted in line graph; *p < 0.05, **p < 0.01, One-way ANOVA test, as compared with initial time point. (**F**) T98G cells stimulated by TNFα (10 ng/ml) for 0, 90, 180 and 360 minutes were harvested and lysed for WB of endogenous ANT1, IκBα and COX-IV. Anti-ANT1 (ab110322; Abcam), anti-IκBα (#4814, CST) and anti-COX-IV (#4850, CST) antibodies were used in WB. β-actin was used as a loading control. Cropped blots were displayed. Values represent means ± SEM (n = 3); **p < 0.01, One-way ANOVA test, as compared with initial time point. (**G**) ANT1 over-expression plasmid (p3xflagANT1) was transfected into T98G cells and stimulated by TNFα for 0, 90, 180 and 360 minutes. Anti-flag (M2, F1804, Sigma-Aldrich) was used to detect exogenous ANT1 expression. Cropped blots were displayed. Values represent means ± SEM (n = 3); NS, no significant difference, One-way ANOVA test, as compared with initial time point.

To examine whether *ANT1* transcription is regulated by NF-κB signalling, RT-PCR was used to measure *ANT1* mRNA levels in HEK293 cells transfected with IKKβ and NF-κB/p65. *ANT1* mRNA was reduced to 60.11 ± 2.307% and 69.14 ± 2.635% by transfection of IKKβ and NF-κB/p65 (*p* = 0.012 and *p* = 0.022, Fig. 1A), while the *ANT1* mRNA level was elevated to 223.9 ± 1.423% by the NF-κB transcriptional activity inhibitor JSH-23 (*p* < 0.01, Bar 2 of Fig. 1B). The mRNA of *ANT1* was also reduced to 39 ± 2.507% by the NF-κB activator H_2_O_2_ (*p* < 0.01, Bar 3 of Fig. 1B). The activation of NF-κB signalling in cells with the transfection of plasmids or stimulators was confirmed by the sharply increased mRNA levels of *IκBα* (~4 fold increase in NF-κB transfected group and ~2 fold increase with H_2_O_2_ treatment) and *TNFα* (~2 fold increase in NF-κB transfected group), which were regarded as the canonical target genes of NF-κB. Consistent with changes in the mRNA level, the ANT1 protein level was decreased to 57.84 ± 8.528% in cells transfected with NF-κB/p65 (*p* = 0.038, Bar 2 of Fig. 1C), and the ANT1 protein level was increased to 122.3 ± 4.273% by NF-κB inhibitor JSH-23 treatment (*p* = 0.037, Bar 3 of Fig. 1C). These results demonstrate that NF-κB signalling represses the transcription of *ANT1*.

To mimic the physiological activation of NF-κB signalling *in vivo*, TNFα was used to stimulate NF-κB signalling in T98G human glioblastoma cells^33^. Electrophoretic mobility shift assay (EMSA) using consensus NRE as a probe showed that nuclear NF-κB activity was markedly elevated after 90 minutes of TNFα (10 ng/ml) treatment and decreased to normal level after 6 hours’ treatment (Fig. 1D). Concomitant with the increased NF-κB activity, real-time PCR showed that *ANT1* mRNA levels in T98G cells were reduced to 49.85 ± 4.496% and 77.83 ± 1.781% after 90 and 180 minutes’ stimulation with TNFα (10 ng/ml) respectively (Fig. 1E). Similarly, ANT1 protein levels were markedly reduced to 16.48 ± 0.5473% and 31.10 ± 1.659% at 90 and 180 minutes of TNFα (10 ng/ml) treatment in T98G cells, and IκBα levels were also reduced to 39.89 ± 5.541% and 16.14 ± 2.718% at 90 and 180 minutes of TNFα (10 ng/ml) treatment. To exclude that changes in ANT1 were not due to mitochondrial content change, COX-IV protein was measured and no changes were observed with TNFα treatment (Fig. 1F). To differentiate between transcriptional level and post-translational level changes in ANT1 protein, exogenous ANT1 protein was expressed by a plasmid vector, and no significant changes were observed in the Western blot (WB) of exogenous ANT1 with anti-flag antibody after simulation of TNFα, indicating that the change in ANT1 by TNFα arose from *ANT1* mRNA transcription and not from post-translational modifications (Fig. 1G). These studies indicate that *ANT1* mRNA and protein are repressed by NF-κB signalling.

### NF-κB suppresses ANT1 transcription by binding to NREs in ANT1 promoter

**Figure 2:**
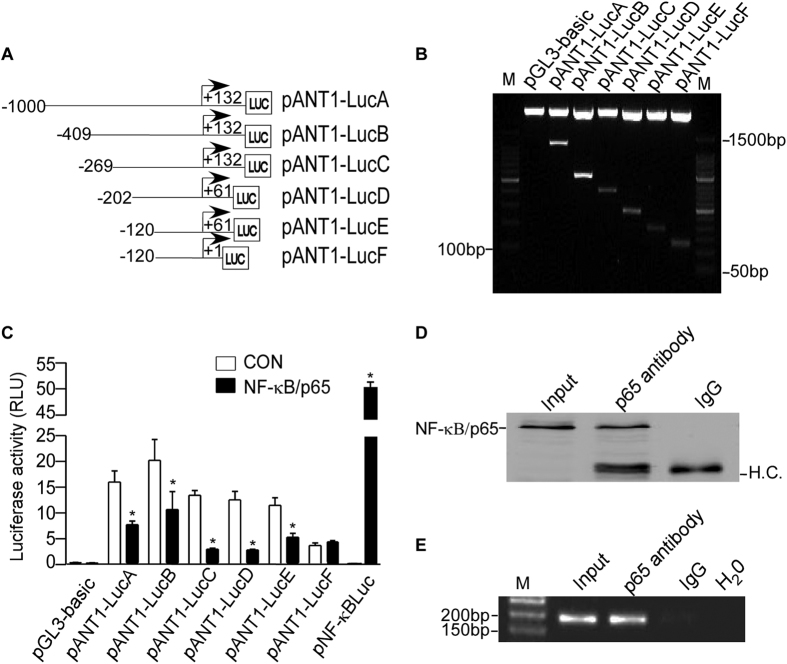
NF-κB negatively regulates the *ANT1* promoter. (**A**) Schematic diagrams showed the ANT1 promoter deletion constructs consisting of serial deletions of 5′-flanking region cloned into the promoterless vector plasmid pGL3-Basic (E1751, Promega) in front of the luciferase reporter gene. Arrow indicates the direction of transcription. The numbers represent the end points of each construct. +1 Was denoted by the first nucleotide of exon 1 in ensemble transcript ENST00000281456.10. (**B**) The deletion plasmids were confirmed by sequencing and restriction enzyme digestion on a 1.5% agarose gel. Vector size is 4.7 kb, and the *ANT1* gene 5′-flanking fragment inserts ranged from 0.1 kb to 1.1 kb. M, marker. (**C**) HEK293 cells were co-transfected with NF-κB/p65 expression vector and various *ANT1* promoter deletion constructs. Plasmid pRL-TK was used to normalise transfection efficiency, and dual luciferase activities were measured at 24 hours by a luminometer (GloMax 20/20 Luminometer, Promega, Wisconsin, USA). The histogram depicts the mean ratios of relative luciferase units of deletions to pGL3-Basic ± SEM (n = 4); *p < 0.05, Student’s t test. RLU, relative luciferase unit. (**D**) Anti-NF-κB/p65 (#8242; CST) was used to immunoprecipitate the cross-linked NF-κB/p65-DNA complex in ChIP assay in HEK293 cells. The immunoprecipitates were analysed with Anti-NF-κB/p65 antibody. IgG was used as negative control. H.C., heavy chain. Cropped gels were displayed. (**E**) A pair of primers was used to amplify *ANT1* promoter region in ChIP. Signals amplified from input were used as size markers for PCR. IgG and H_2_O were used as negative controls. Lane 1 is input. Lane 2 is immunoprecipitate by anti-NF-κB/p65 antibody. Lane 3 is immunoprecipitate by IgG antibody and Lane 4 uses H_2_O as a blank template control. M, marker. Cropped gels were displayed.

To further clarify the molecular mechanism of *ANT1* transcription, we cloned an 1132-bp (−1000 to +132 bp) fragment located in the 5′-flanking region of the human *ANT1* gene (Fig. 2A and B) into promoterless vector pGL3-Basic. +1 was denoted by the first nucleotide of exon 1 in ensemble transcript ENST00000281456.10. The promoter construct showed a high luciferase activity, indicating that the region −1000 + 132 bp contained the functional promoter region of the human *ANT1* gene. As expected, the luciferase activity of *ANT1* promoter (pANT1-LucA) reflected by dual-luciferase assay was significantly lower in NF-κB/p65 transfected cells than in controls (6.814 ± 0.6215 RLU compared with 14.28 ± 1.919 RLU), indicating that the 1.1-kb fragment contained NF-κB responsive elements. To identify the location of NREs, five truncated pANT1-Luc (B-F) were constructed, containing different regions of *ANT1* gene promoter. Dual-luciferase activity showed that NF-κB/p65 decreased the activity of *ANT1* promoters, with the exception of pANT1-LucF (Fig. 2C), indicating that the putative NREs may be located from +1 to +61 bp. Chromatin immunoprecipitation (ChIP) showed that NF-κB/p65 antibody specifically pulls down a genomic DNA region of −74 bp to +108 bp containing the putative NREs (Fig. 2D and E).

**Figure 3:**
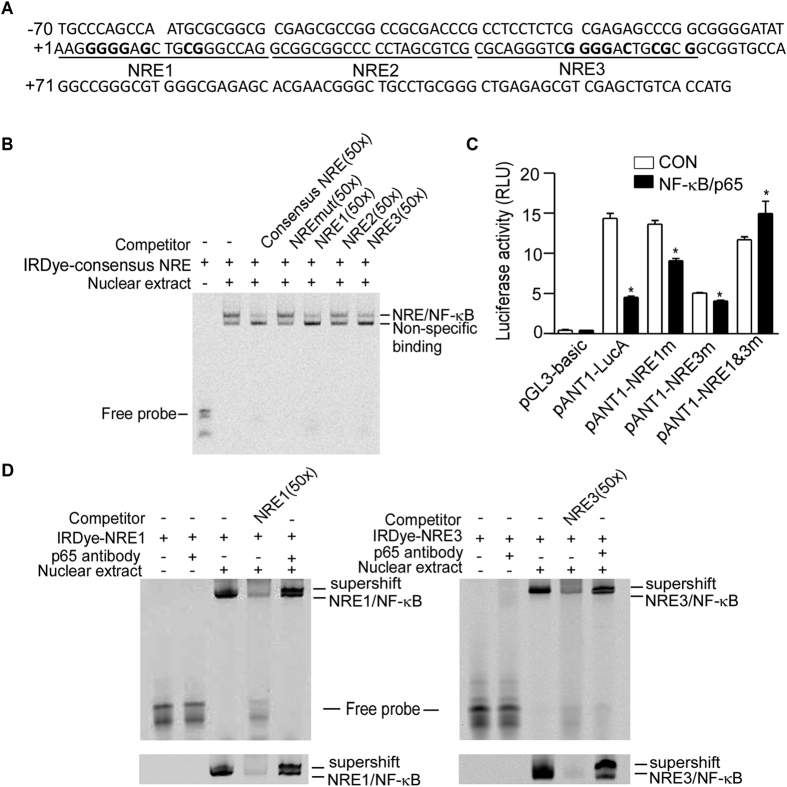
Identification of the functional NRE sites in *ANT1* gene promoter. (**A**) The nucleotide sequence was from −70 bp to +121 bp region of ANT1 promoter containing the three putative NRE sites. Those bold bases depict the mutant sites in our NRE1 and NRE3. NRE1 site (5′-AAG**GGGG**A**G**CTG**CG**GGCCAG-3′) was mutated to NRE1 mutant (5′-AAG*aaac*A*a*CTG*tt*GGCCAG-3′) and NRE3 (5′-CGCAGGGTC**GGGG**A**C**TG**CG**C**G**-3′) was mutated to NRE3 mutant (5′-CGCAGGGTC*aaca*A*a*TG*tt*C*a*-3′). (**B**) EMSA was performed with IRDye 800-labelled consensus NRE oligo. Competition assays were performed by unlabelled consensus NRE, mutant consensus NRE and three putative NREs from *ANT1* promoter (50x). NRE, NF-κB responsive elements. (**C**) HEK293 cells were co-transfected with NF-κB/p65 expression vector and pANT1-NRE1mut, pANT1-NRE3mut or pANT1-NRE1&3mut. The Renilla luciferase vector pRL-TK was used to normalize transfection efficiency. And dual luciferase activities were measured 24 hours after transfection by a luminometer (GloMax 20/20 Luminometer, Promega, Wisconsin, USA). The histogram depicts the mean ratios of relative luciferase units of mutants to pGL3-Basic ± SEM (n = 4); *p < 0.05, Student’s t test. RLU, relative luciferase unit. (**D**) IRDye 800-labelled NRE1 or NRE3 oligonucleotides from *ANT1* promoter were used as probes in EMSA. The addition of anti-NF-κB/p65 antibody further shifted the NREs-NF-κB complex band to a higher molecular weight (super-shift). The results of longer time running of EMSA (cropped) were shown below to make the super-shift much clear.

Bioinformatic analysis of *ANT1* promoter region of −74 bp to +108 bp using JASPAR software (JASPAR 2016)^34^ revealed three putative NREs located at +2 bp to +14 bp, +20 bp to +32 bp and +49 bp to +61 bp. To further identify the putative NREs, three putative NREs (NRE1, NRE2 and NRE3) were synthesised, spanning the region from +1 to +61 bp of *ANT1* promoter (Fig. 3A). EMSA was performed using the consensus NRE as a probe and 50x excess of the three putative *ANT1* NREs as competitors. The NRE/NF-κB band (Lane 2 of Fig. 3B) could be out-competed using the cold consensus NRE (Lane 3 of Fig. 3B), as well as NRE1 and NRE3 spanning from +1 to +20 bp and +41 to +61 bp (Lanes 5 and 7 of Fig. 3B). Competitors using the mutant consensus NRE or the NRE2 spanning from +21 to +40 bp could not out-compete the shifted band (Lanes 4 and 6 of Fig. 3B). In addition, the single mutation of either NRE1 or NRE3 in the pANT1-lucA construct did not abolish the effect of NF-κB in the *ANT1* promoter, while the dual mutations abolished the repressing effects of NF-κB on the *ANT1* promoter, indicating that the *ANT1* promoter contains two NREs at +1 bp to +20 bp and +40 bp to +61 bp (Fig. 3C). Furthermore, oligonucleotide probes using the *ANT1* gene promoter NRE1 and NRE3 sites were synthesised and end-labelled with IRDye 800 infrared dye. A shifted NRE/NF-κB band was observed after addition of nuclear extract (Lane 3 of Fig. 3D), and the shifted band was further shifted to a greater molecular weight after addition of anti-NF-κB antibody, suggesting the specificity of NRE and NF-κB binding complexes (Fig. 3D). The results of longer time running of EMSA were shown below to make the super-shift much clear. Taken together, these results demonstrate that the NF-κB transcription factor binds to the two NREs in *ANT1* promoter at +1 to +20 bp and +41 to +61 bp.

### NF-κB lowers the ATP/ADP exchange rate and ATP level through ANT1

**Figure 4:**
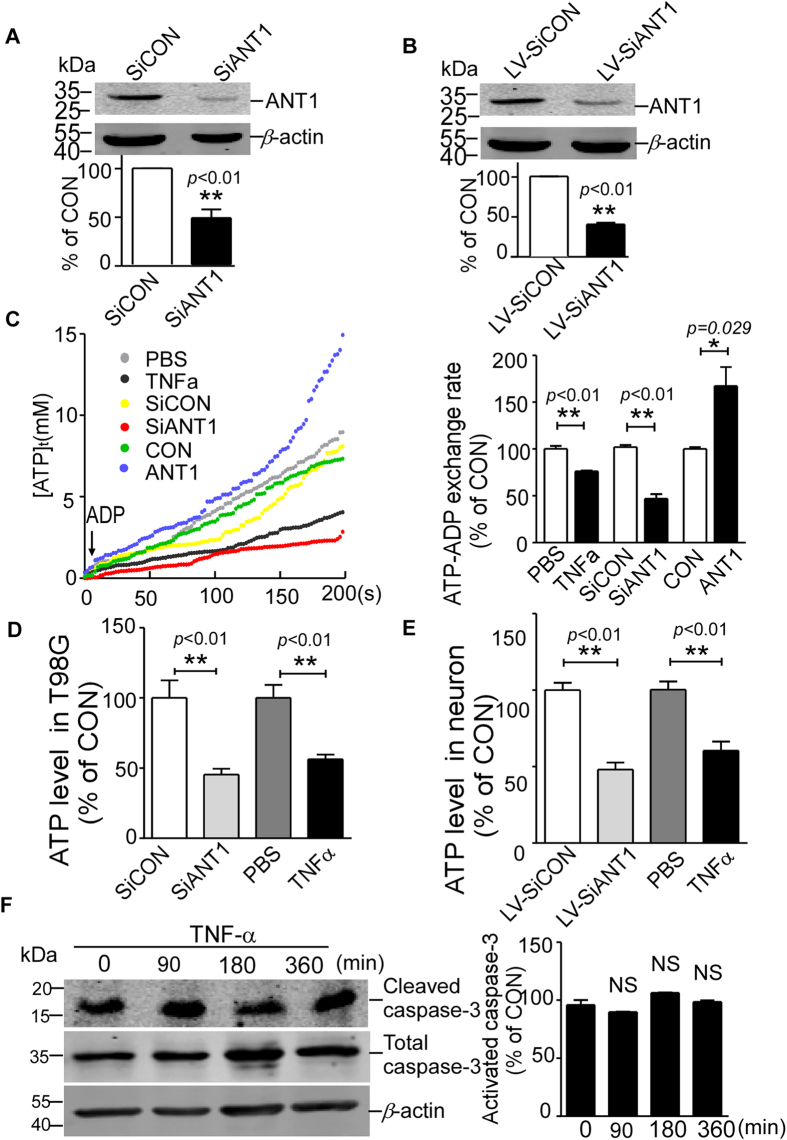
NF-κB impairs mitochondrial ATP/ADP exchange rate and intracellular ATP level. (**A**,**B**) T98G cells were transfected with pSiANT1 and primary neurons were infected with lentivirus LV-SiANT1 or negative control (pSiCON or LV-SiCON) for 72 hours. Cell lysates were separated by SDS-PAGE and analysed with anti-ANT1 antibody (ab110322; Abcam). Cropped blots were displayed. The histogram depicts the mean ratios of ANT1 protein to β-actin ± SEM (n = 3); **p < 0.01, Student’s t test. (**C**) ATP/ADP exchange rates were determined in T98G cells with TNFα treatment, ANT1 knockdown (pSiANT1 transfected) and ANT1 over-expression (pANT1 transfected). ANT1 knockdown and TNFα treatment decreased ATP/ADP exchange rate while ANT1 overexpression increased the rate. The histogram depicts the means of the ATP/ADP exchange rates in treatment groups ± SEM (n = 3); *p < 0.05, **p < 0.01, Student’s t test, as compared to their respective control groups (CON). (**D**,**E**) ATP levels in T98G (**D**) and neurons (**E**) were measured by a luminometer using an ATP determination kit (A22066; Invitrogen). The histogram depicts the means of the cellular ATP level in treatment groups ± SEM (n = 3); ** p < 0.01, Student’s t test, as compared to their respective control groups (CON). (**F**) Total and cleaved caspase-3 were determined by caspase-3 (#9665, CST) and cleaved caspase-3 (#9664, CST) antibodies in T98G cells treated by TNFα (10 ng/ml) for 0, 90, 180 and 360 minutes. Cropped blots were displayed. The histogram depicts the mean ratios of cleaved caspase-3 protein level to total caspase-3 protein level ± SEM (n = 3); NS, no significant difference, One-way ANOVA test, as compared with initial time point.

Our data show that the gene transcription of *ANT1* may be negatively regulated by NF-κB signalling. Because the primary function of ANT1 is the exchange of cytosolic ADP and intramitochondrial ATP, we then examined whether NF-κB modulates the ANT1-dependent ATP/ADP exchange rate and subsequently affects ATP production. The ANT1 knockdown effects of plasmid pSiANT1 (SiANT1) and lentivirus SiANT1 (LV-SiANT1) were validated in T98G and neurons (Fig. 4A and B). T98G cells were treated with TNFα (10 ng/ml) for three hours to activate NF-κB signaling. The data show that the ATP/ADP exchange rate in TNFα treated T98G cells was reduced to about 75% of the control cells (*p* < 0.01, Bar 1 vs. Bar 2 of Fig. 4C). The exchange rate was reduced to 45.28 ± 3.937% in ANT1 knockdown cells (*p* < 0.01, Bar 3 vs. Bar 4 of Fig. 4C). In addition, the exchange rate was increased to 167.4 ± 20.21% in ANT1 over-expressed cells (*p* = 0.029, Bar 5 vs. Bar 6 of Fig. 4C). Consistent with the reduction in ATP/ADP exchange rate, ATP levels were reduced to 45.46 ± 4.092% in T98G cells transfected with pSiANT1 (*p* < 0.01, Bar 1 vs. Bar of Fig. 4D). And ATP levels were reduced to 48.11 ± 4.552% in primary rat neurons infected with LV-SiANT1 (*p* < 0.01, Bar 1 vs. Bar 2 of Fig. 4E). Similarly, TNFα treatment decreased ATP production in T98G cells to 56.39 ± 3.269% (*p* = 0.004, Bar3 vs. Bar 4 of Fig. 4D). And TNFα treatment decreased ATP production in primary neurons to 60.43 ± 6.021% (*p* = 0.001, Bar 3 vs. Bar 4 of Fig. 4E). There was no change in caspase-3 activation in T98G cells after TNFα treatment, indicating that the reduction of ATP had nothing to do with apoptosis (Fig. 4F). All these results indicate that NF-κB activation by TNFα treatment may diminish the ATP/ADP exchange rate and reduce the ATP level through regulating the *ANT1* gene expression.

### NF-κB decreases the Ca^2+^-induced mPTP opening level via ANT1

**Figure 5:**
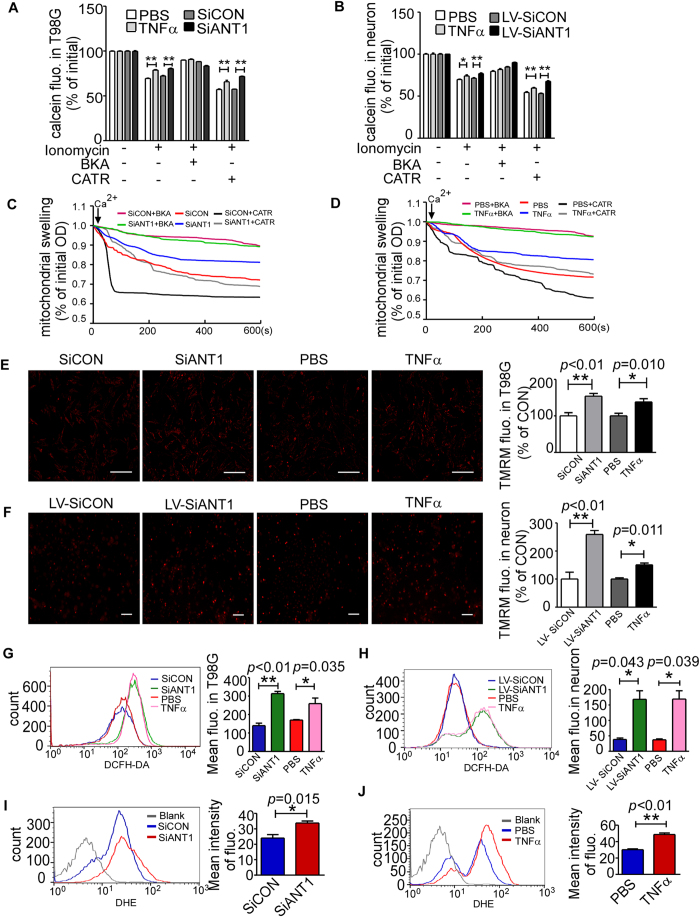
NF-κB decreased Ca^2+^-induced mPTP opening, increased Δψ_m_ and ROS. (**A**,**B**) To determine mPTP opening, cells in different treatment groups were loaded with calcein-AM for 30 minutes and subsequently incubated with ionomycin (5 μM), BKA (5 μM) or CATR (1 μM) for another 30 minutes. The mPTP opening level was measured as calcein loss by Varioskan flash instruments (Thermo Scientific, USA). The histogram depicts the means of remaining calcein fluorescences of treatment groups ± SEM (n = 4); *p = 0.028 **p < 0.01, Student’s t test, as compared with their respective control. (**C**,**D**) Ca^2+^-induced mitochondrial swelling was assayed by the decrease in absorbance at 540 nm and the curves represent typical recordings from experiments of at least three different mitochondrial preparations. (**E**,**F**) Δψ_m_ in T98G cells (**E**) and neurons (**F**) was determined by TMRM (50 nM) staining using a fluorescence microscope (Licor, USA) and analyzed by Image J software (Image J 1.46r); *p < 0.05 **p < 0.01, Student’s t test, as compared with their respective control. Scar bar = 200 μm. (**G**,**H**) ROS production was measured using DCFH-DA (5 μM) fluorescence in both T98G and neurons by a flow cytometer (FACS, BD Aria III). The histogram depicts the means of TMRM fluorescence of treatment groups ± SEM (n = 3); *p < 0.05, **p < 0.01, Student’s t test, as compared with their respective controls. (**I**,**J**) Superoxide radical**s** in T98G was determined by DHE (5 μM) staining, and the fluorescence detected by a flow cytometer (FACS, BD Aria III). The histogram depicts the means of DHE fluorescence of treatment groups ± SEM (n = 3); *p < 0.05, **p < 0.01, Student’s t test, as compared with their respective controls.

ANT1 had been identified as being responsible for Ca^2+^-induced mPTP opening, and it has been reported that more Ca^2+^ than usual is required to activate the mPTP of mitochondria lacking ANT1^35^,^36^. To verify whether the Ca^2+^-induced mPTP opening level is altered by NF-κB signalling, T98G cells were treated with the TNFα (10 ng/ml) for three hours, or transfected by pSiANT1. Primary neurons were treated with TNFα (10 ng/ml) for three hours or transfected by lentivirus LV-SiANT1. Ca^2+^ ionophore ionomycin (5 μM) was used to trigger mPTP opening. mPTP opening is reflected by the decreased percentage of initial calcein fluorescence. The basal level of calcein fluorescence showed no difference in either T98G or neurons (Bars 1–4 of Fig. 5A, and Bars 1–4 of Fig. 5B). With the treatment of ionomycin, calcein fluorescence was less reduced by TNFα treatment or ANT1 knockdown than the controls (Bars 5–8 of Fig. 5A, and Bars 5–8 of Fig. 5B). There was no change in the levels of calcein fluorescence in T98G or neurons after treatment with bongkrekate (BKA), an inhibitor of Ca^2+^-induced mPTP opening (Bars 9–12 of Fig. 5A, and Bars 9–12 of Fig. 5B). In addition, with treatment of carboxyatractylate (CATR), an activator of Ca^2+^-induced mPTP opening, TNFα and ANT1 knockdown also showed less decrease of calcein fluorescence compared with the control (Bars 13–16 of Fig. 5A, and Bars 13–16 of Fig. 5B). To confirm this result, a simple measurement of mitochondrial swelling upon Ca^2+^ overload were used in T98G. Transfected or TNFα treated T98G cells were added with CATR or BKA, and Ca^2+^-induced mitochondrial swelling was assayed by the decrease in absorbance at 540 nm. TNFα and ANT1 knockdown also showed less level of mitochondrial swelling than their respective controls (Fig. 5C and D). Taken together, these data imply that NF-κB activation by TNFα treatment may decrease the Ca^2+^-induced mPTP opening level via ANT1 expression.

### NF-κB increases mitochondrial membrane potential (Δψ_m_) and ROS production

The mitochondria in ANT1-deficient neurons increases mitochondrial membrane potential (Δψ_m_), indicating an inherent regulation between ANT1 and Δψ_m_^37^. To elucidate whether NF-κB signalling may affect the Δψ_m_ via ANT1, mitochondrial Δψ_m_ were examined in T98G cells and primary neurons treated with TNFα or transfected by pSiANT1 and lentivirus LV-SiANT1. ANT1 knockdown and TNFα (10 ng/ml) treatment increased Δψ_m_ level in T98G cells by ~1.5 fold or ~1.3 fold (*p* < 0.05, Bar 1 vs. Bar 2, and Bar 3 vs. Bar 4 of Fig. 5E). ANT1 knockdown and TNFα (10 ng/ml) treatment elevated the Δψ_m_ by ~2.6 fold or ~1.5 fold in primary neurons (*p* < 0.05, Bar 1 vs Bar 2, and Bar 3 vs. Bar 4 of Fig. 5F). We next determined the cellular ROS, since alterations in mitochondrial membrane potential usually induce ROS generation. In T98G cells, the ROS was increased by ~1.6 fold or ~2.3 fold by TNFα (10 ng/ml) treatment and ANT1 knockdown (*p* < 0.05, Bar 1 vs. Bar 2, and Bar3 vs. Bar 4 of Fig. 5G). And ROS in primary neurons were increased by ~4.0 fold and ~4.4 fold after TNFα (10 ng/ml) treatment and ANT1 knockdown by lentivirus LV-SiANT1 (*p* < 0.05, Bar 1 vs. Bar 2, and Bar 3 vs. Bar 4 of Fig. 5H). To confirm this, the production of superoxide radicals was examined by dihydroethidium (DHE) staining in T98G. ANT1 knockdown and TNFα also showed ~1.5 fold and ~2 fold increase in the mean intensity of DHE fluorescence (*p* < 0.05, Bar 1 vs. Bar 2, in 5I and Bar 1 vs. Bar 2 of Fig. 5J). These results indicate that ROS are increased by NF-κB signalling through reduction of ANT1.

## Discussion

NF-κB is a transcriptional regulator involved in many pivotal roles in cellular functions including inflammatory responses, tumorigenesis and apoptosis. Our study here identified *ANT1* as a novel target of NF-κB signalling. We showed that NF-κB/p65 decreased *ANT1* mRNA and protein expression by binding to the two NREs located at +1 bp to +20 bp and +41 bp to +61 bp of the *ANT1* promoter. Furthermore, NF-κB signalling affected the normal mitochondrial function relating to ANT1, including mitochondrial ATP/ADP exchange, calcium-induced mPTP opening and mitochondrial membrane potential, and subsequently led to impairment of ATP production and over-generation of reactive oxygen species. Our study of interactions of NF-κB and ANT1 therefore reveals a molecular linkage between inflammation and mitochondrial dysfunction. Previous study showed that ANT1 was decreased in inflammatory heart in mice and ANT1 knockdown increased swollen mitochondria and mitochondrial ROS^31^. Our study here provided a molecular mechanism in inflammation induced mitochondrial dysfunctions.

Upon activation of NF-κB signalling by such as TNFα treatment, the IκBα serine residues 32/36 will be phosphorylated by IKK and subjected to ubiquitination and degradation by proteasome, NF-κB is translocated into nucleus and downregulates *ANT1* gene transcription. IκBα, the inhibitor and cytoplasmic partner of NF-κB, has been shown to physically interact with ANT1 in mitochondrial intermembrane space, and IκBα·NF-κB complex may exert a transcriptionally independent regulatory function on apoptosis^38^. And it may be possible that NF-κB could affect ANT1 function through a protein-protein interaction with ANT1. Whether downregulation of ANT1 by NF-κB affects IκBα·NF-κB complex localization and function in mitochondria remains unknown though IκBα localization in mitochondria is not affected by its phosphorylation or degradation^38^. Overexpression of ANT1 recruits IκBα·NF-κB complex into mitochondria and decreases NF-κB transcriptional activity in nucleus^39^, which may further affect *ANT1* transcription and leads to increased *ANT1* expression. There are several levels of interactions between *ANT1* and NF-κB in nucleus and in mitochondria. Over a certain level of *ANT1* expression, a vicious cycle between ANT1 and NF-κB may effect to facilitate cell death. Consistent with this consumption, ANT1 overexpression in cells can dominantly induce cell apoptosis^19^.

Our results showed ANT1 reduction by NF-κB or knockdown reduced ATP/ADP exchange rate and ATP production. The reduction of ATP in cells with ANT1 knocked down or reduced by NF-κB activation was probably due to the decreased ADP levels in mitochondria. The reduction of ATP may not be a result of mitochondrial apoptosis signalling, since the mitochondrial inner membrane potential was increased concomitantly. In addition, caspase-3 activation was not changed by TNFα, indicating no apoptosis was induced though there was a significant reduction of ATP. TNFα has also been shown to reduce ATP production by inhibiting oxidative phosphorylation through tyrosine phosphorylation of cytochrome c oxidase^27^. Our studies here suggested ATP can also be reduced due to reduction of ANT1 transcription by TNFα.

Mitochondria are required for cellular bioenergetics, and their dysfunction has been considered as a central cytopathology of neurodegenerative diseases including Alzheimer’s disease and Parkinson’s disease^40^. Our study here implies that neuroinflammation, a key feature in neurodegenerative diseases, may lead to dysregulation of ANT1 protein in mitochondria and dysfunctions of mitochondria that in turn leads to excessive ROS production. ROS is normally produced in the mitochondrial electron-transport chain (ETC) during respiration, and excess ROS may damage cellular lipids, proteins and DNA, inhibiting their normal functioning^41^. Our finding that NF-κB/p65 may elevate ROS levels, as down-regulated ANT1 does, is also supported by reports of increased ROS production and oxidative stress in hearts of ANT1 knockout mouse^42^. Recent studies have shown mitochondrial ROS can drive proinflammatory cytokine productions including IL-1β, IL-6 and TNF, resulting in activation of caspase-1-activating complexes known as inflammasomes^43^,^44^. Human disease with chronic inflammation such as Crohn’ disease, diabetes and atherosclerosis are also characterized with excessive ROS^43^. The harmful positive feedback loop between inflammation and ROS induces excessive cell and tissue damage and ultimately leads to destruction of normal tissue and chronic inflammation. Our study here implicated the *ANT1* gene is regulated by inflammation and ANT1 reduction successively induces ROS production, thus providing a molecular linkage between inflammation and ROS.

## Materials and Methods

### Materials

ANT1 mAb (ab110322, Abcam, Cambrige, UK); ATP Determination Kit (A22066, Invitrogen, Waltham, USA); anti-flag mAb (M2, F1804, Sigma-Aldrich, Saint Louis, USA);*β*-actin mAb (SAB1403520; Sigma-Aldrich, Saint Louis, USA); Bongkrekic acid (1820–100, Biovision, USA); calcein-AM (17783, Sigma-Aldrich, Saint Louis, USA); carboxyatractyloside (C4992, Sigma-Aldrich, Saint Louis, USA); Chromatin Immunoprecipitation (ChIP) Assay Kit (17–295, Milllipore, Darmstadt, Germany); cleaved caspase-3 mAb (#9664, CST, Beverly, USA); CoCl_2_ (V900021, Sigma-Aldrich, Saint Louis, USA); COX-IV mAb (#4850, CST, Beverly, USA); DCFH-DA (S0033, Beyotime, Nanjing, China); Dihydroethidium (DHE, S0063, Beyotime, Nanjing, China), digitonin (D141, Sigma-Aldrich, Saint Louis, USA); Dual-Luciferase^®^ Reporter Assay System (E1910, Promega, Wisconsin, USA);Dulbecco’s modified Eagle’s medium (SH30243.01B, Hyclone, South Logan, USA); Fetal bovine serum (10100147, Gibco, Gaithersburg, MD); IκB-α mAb (#4814, CST, Beverly, USA); ionomycin (S1672, Beyotime, Nanjing, China); JSH-23 (S7351;Selleckchem, Houston, USA); Lipofectamine^TM^ 2000 transfection reagent (11668027, Invitrogen, Waltham, USA); Magnesium Green™ (M3733, Invitrogen, Waltham, USA); NF-κB/p65 mAb (#8242, CST, Beverly, USA); Odyssey EMSA Buffer Kit (ABIN2169587, Li-cor, USA); Opti-MEM(51985042, Gibco, Gaithersburg, MD);PEG8000 (89510, Sigma-Aldrich, Saint Louis, USA); secondary antibodies (Jackson Immuno Research, West Grove, USA);SYBR-Green PCR Master Mix (QPK-201, Toyobo, Japan); TMRM, (T668, Invitrogen, Waltham, USA); TNFα (10602-HNAE-10, Sino Biological lnc. Beijing, China); total caspase-3 mAb (#9665, CST, Beverly, USA); TRI reagent (T9424, Sigma-Aldrich, Saint Louis, USA).

### Cell cultures

HEK293, HEK293T and T98G human glioblastoma cells were cultured in high glucose Dulbecco’s modified Eagle’s medium supplemented with 10% FBS. Neuronal cells for primary cultures were from Wistar rat embryos at 17–18 days as described previously^45^. All cells were maintained at 37 °C in an incubator containing 5% CO_2_. The experimental protocols were approved by the Animal Care and Protection Committee of Shandong University and institutional Ethics Committees of Qilu Hospital, and in compliance with ARRIVE guideline.

### Real-time quantitative RT-PCR mRNA

TRI reagent was applied to extract total RNA from cells. Real-time RT-PCR was carried out on ABI 7900HT Fast Real-Time PCR System (Foster City) with SYBR-Green PCR Master Mix. A comparative CT method (2^−ΔΔCT^) was used to analyse the gene expression level. Primers for semi-quantitative and real-time quantitative PCR were as follows: human ANT1-202F:5′-CTCTCCTTCTGGAGGGGTAAC-3′ANT1-327R:5′-GAACTGCTTATGCCGATCCAC-3′; human *β*-actin-F 5′-GACAGGATGCAGAAGGAGAT-3′,*β-*actin-R 5′-TGATCCACATCT-GCTGGAAGGT-3′.

### Plasmids construction

Plasmids encoding IKKβ and NF-κB/p65 were purchased from Vigene Biosciences (Jinan, China). The plasmid pNF-κB-Luc contains three NF-κB responsive elements before the minimal IL-2 promoter, followed by the firefly luciferase (luc) gene and it was designed to monitor the activation of NF-κB signal transduction pathway^46^. The 5′ upstream region of human ANT1 gene (−1001 to +132, pANT1-LucA) was obtained by PCR of genomic DNA isolated from BAC-human-rp4 using a pair of primers ANT1-LucA-F (5′-CCGCTCGAGTATTTGAAAACCTGTATGGT-3′) and ANT1-LucA-R (5′-CCCAAG- CTTGGTGACAGCTCGACGCTCTCAG-3′) and subcloned into pGL3-Basic vector (E1751, Promega, Wisconsin, USA) using *Xho1* and *HindIII*. pANT1-LucB was obtained through the digestion of pANT1-LucA using *SacI*. pANT1-LucC was obtained by PCR using ANT-LucC-F (5′-CCGCTCGAGCCTAAGGCTGCCTGTGCTATAAATA-3′) and ANT1-LucA-R. pANT1-LucD was obtained by PCR using ANT1-LucD-F (5′-CCGCTCGAGGCGGGACAGATAACATGAATGTGCCC-3′) and ANT1-LucD-R (5′-CCCAAGCTTCGCGCAGTCCCCGACCCTGCGCGACGCT-3′). pANT1-LucE was obtained by PCR using ANT1-LucE-F (5′-CCGCTCGAGCCTTCGCCCCCGATGCCCTC-3′) and ANT1-LucD-R. pANT1-LucF was obtained by PCR using ANT1-LucE-F and ANT1-LucF-R (5′-CCCAAGCTTATATCCCCGCCGGGCTCTCGCGAGAGGA-3′). pANT1-NRE1m was obtained by PCR using ANT1-LucA-F, NRE1m-MS (5′-CGGGGATATAAG*aaacaa*CTG*tt*GGCCAGGCGGCGG-3′) and NRE1m-MAS(5′-CCGCCGCCTGGCC*aa*CAG*ttgttt*CTTATATCCCCG-3′). pANT1-NRE3m was obtained by PCR using ANT1-LucA-F and NRE3m-HinR (5′-CCCAAGC-TTTGAACATTTGTTGACCCTGCGCGACGCTAGG-3′). Full-length *ANT1* cDNA was subcloned in p3XFLAG-CMV^™^-10 vector (E7658; Sigma-Aldrich, USA) using *EcoRV* and *BamH1*. Plasmid pSiANT1 containing human *ANT1* siRNA was generated using a RNAi vector pSUPER (VEC-PBS-0002; OligoEngine, Seattle, USA) as described previously^47^ and the target sequence for human *ANT1* siRNA is 5′-GCAGTACAAAGGGATCATTGA-3′.

### Plasmids sequencing

All the constructed plasmids were confirmed by DNA sequencing. Our deletions of plasmids were sequenced by forword primer RVP3 (5′-CTAGCAAAATAGGCTGTCCC-3′) supported by BioSune Biotechnology Inc. (Jinan, China).

### Lentivirus packaging

*ANT1* siRNA were dual-enzyme digested by *EcoRI* and *ClaI* from pSiANT1 and subcloned into lentiviral vector pLVTHM a gift from Didier Trono Lab (Addgene plasmid #12247). In order to avoid the interference of green fluorescent protein from pLVTHM, the GFP tags in pLVTHM-siANT1 and pLVTHM were digested by *PmeI* and *NdeI*. 4 × 10^6^ HEK293T cells were plated one day before the transfection of pLVTHM-siANT1 and empty vector pLVTHM together with packaging plasmids VSVG (Trono Lab, Addgene plasmid #12259) and psPAX2 (Trono Lab, Addgene plasmid #12260) using the lipofectamine^TM^ 2000. The media was changed by serum free media after 8 hours of transfection. The supernatant containing viral particles were collected at 48 and 72 h after transfection, filtered through a 0.45 μm filter, concentrated by PEG8000 overnight at 4 °C. After centrifugation twice at 4,000 rpm for 5 minutes, the viral particles were resuspended by 100 μl Opti-MEM and aliquots were stored at −80 °C.

### Chromatin immunoprecipitation (ChIP) and electrophoretic mobility shift assay (EMSA)

ChIP and EMSA were performed as previously described^48^. The association of exogenous NF-κB with ANT1 promoter in HEK293 cells was confirmed using a chromatin immunoprecipitation assay kit # 17-295, Millipore) following the manufacturer’s protocol. Transfected by NF-κB/p65 plasmid, cells each about 5 × 10^6^ cells) were cross-linked by formaldehyde (final concentration of 1%) for 10 min at 37 °C, and then washed by cold PBS twice. The cells were centrifuged and pellets were lysed by 100 μl 1% SDS lysis buffer and sheared by sonication. The sonicated cell supernatant was then diluted by 9 fold (900 μl) ChIP dilution buffer and 1/50 (20 μl) was accepted as input. After the cross-linked proteins and DNA pulled down with p65 antibody (1:100, #8242, CST) or normal IgG as a negative control overnight at 4 degree, the cross links (both input and immunoprecipitated group) were reversed and DNA was recovered by phenol/chloroform extraction. The extract ed DNA was re-dissolved in 20 μl PCR-grade water. 1 μl of input or immunoprecipitated DNA were used as templates and confirmed by PCR (35 cycles) using following promoter primers (ChIP-F:5′-CACCTGCCCAGCCAATGC-3′ and ChIP-R: 5′-CGCAGGCAGCCCGTTCGT-3′). Products of ChIP-PCR were separated on a 1% agarose gel with ethidium bromide. Immunoprecipitation of proteins, after ChIP with antibodies against NF-κB/p65, was confirmed by Western blot analysis before the ChIP-PCR analysis. For EMSA assay, infrared dye-labeled probe (50 nM) were used in respective incubation and the three sense sequences of *ANT1* NREs were 5′-AAGGGGGAGCTGCGGGCCAG (NRE1), 5′-GCGGCGGCCCCCTAGCGTCG (NRE2) and 5′-CGCAGGGTCGGGGACTGCGCG (NRE3). Consensus NRE and mutant NRE were 5′-AGTTGAGGGGACTTTCCCAGGC, 5′-CAAAAATGTTCAAAAATGTT.

### ATP level measurement

ATP level was determined according to a method developed by Yang *et al*.^49^ and measured by a bioluminescence assay using ATP determination kit (A22066; Invitrogen, Waltham, USA) as indicated by the manufacturer. Briefly, cultured cells were counted. 5 × 10^5^ cells were collected with a centrifugation at 800 g for 5 min and suspended in 500 μl boiling water. The cell pellets were then centrifuged at 12,000 *g* for 10 min and the supernatants were collected. We used the 10 μl of supernatants per assay in a final reaction volume of 100 μl. Bioluminescence of ATP was acquired by a luminometer (20/20, Promega Glomax). Luciferase values were converted to nanomoles of the amount of ATP by plotting against a standard curve with certain concentrations of ATP (1 nM to 1 μM).

### Analysis of ATP-ADP exchange rate

The ATP-ADP exchange rate was measured as previously described^50^,^51^. ADP-ATP exchange rate mediated by the ANT1 was determined with the addition of BeF^3−^ and Na_3_VO_4_ to media of digitonin-permeabilized T98G cells. Cells were cultured in 12-wells plates, suspended in the buffer (8 mM KCl, 110 mM K-gluconate, 10 mM NaCl, 10 mM Hepes, 10 mM KH_2_PO_4_, 5 μM EGTA, 10 mM Mannitol, 25 μM AP_5_A, 5 mM NaF, 0.2 mM BeSO_4_, 30 μM Na_3_VO_4_, 5 μM EDTA and 0.5 mg/ml bovine serum albumin (fatty acid-free), pH 7.25) and treated by 50 μM digitonin. After the addition of ADP (2 mM) and Magnesium Green K+ salt (1 μM), a fluorescent magnesium indicator, the rate of ATP appearing in the medium was calculated from the measured rate of change in free extra-mitochondrial Mg2+. The assay is designed such that all ATP-ADP utilizing reactions were inhibited with the exception of the ANT1 mediated changes in [Mg2+] free in the extra-mitochondrial volume, as a result of ADP-ATP exchange. Magnesium Green fluorescence was recorded in Varioskan flash instruments (Thermo Scientific, USA) respectively using 505 and 535 nm excitation and emission wavelengths. Experiments were performed at 37 °C.

### Determination of mPTP opening

Cells cultured in 24-wells plate were collected and incubated with calcein-AM (2 μM) in a medium (containing 120 mM NaCl, 5.0 mM KCl, 2.0 mM CaCl_2_, 20 mM HEPES and 15 mM glucose at pH 7.4) at 37 °C for 30 min, which penetrates into the cytoplasm and mitochondria. And the fluorescence from cytosolic dye was quenched by the addition of CoCl_2_ (1 mM) for another 30 minutes. After washing cells for three times, half of the cells were left as is, and the other half were treated with 500 μM CaCl_2_, Ca^2+^ ionophore ionomycin (5 μM), CATR (1 μM) or BKA (5 μM) to trigger or inhibit mPTP opening for another 30 minutes. Then the calcein fluorescence was detected by Varioskan flash instruments (Thermo Scientific, USA) described by Petronilli^52^. And the decreased percentage of initial calcein fluorescence could be accepted as mPTP opening level. For determination of mitochondrial swelling, the isolated mitochondria in T98G cells were used to measure the mitochondrial swelling on Ca^2+^ overload. Briefly, cells (about 10^7^ cells) were washed by PBS at room temperature for twice and collected after the centrifugation at 1000 g for 5 min. Then the cell pellets were suspended in ice-cold buffer (150 mM MgCl_2_, 10 mM KCl, 25 mM Tris HCl, 1 mM EDTA, 0.25 M Sucrose, PH 7.4) containing protease and phosphatase inhibitor cocktail, homogenized and centrifuged at 1000 g for 10 min at 4 °C. The supernatants were centrifuged at 8000 g for 15 min at 4 °C and the resulted pellets contained the mitochondrial fractions. Then the pellets were suspended in mitochondrial swelling buffer (125 mM KCl, 2 mM K_2_HPO_4_, 1 mM MgCl_2_, 20 mM Hepes, 5 mM glutamate, 5 mM malate and 2 μM rotenone, pH7.4) and 100 μM CaCl_2_ to trigger the mitochondrial swelling. ANT1 ligand CATR (1 μM) or BKA (5 μM) was used to trigger or inhibit mPTP opening. Ca^2+^-induced mitochondrial swelling was assayed by the decrease in absorbance at 540 nm and the curves represent typical recordings from experiments of at least three different mitochondrial preparations.

### Measurement of intracellular ROS

For the determination of ROS, 5 μM dichlorofluorescein diacetate (DCFH-DA) staining was performed using reactive oxygen species assay kit following the manufacturer’s instructions. DCFH-DA was deacetylated intracellularly by nonspecific esterase, which was further oxidized by ROS to the fluorescent compound dichlorofluorescein (DCF). DCF fluorescence was detected by FACScan flow cytometer (FACS, AriaIII). For each sample 30 000 events were collected. The mean DCFH-DA fluorescence intensity was determined using FlowJo software (FlowJo 10.0.7). Transfected or TNFα treated T98G cells cultured in 6-wells plate were harvested and then incubated with 5 μM Dihydroethidium (DHE, S0063, Beyotime) for 45 min at 37 °C in the dark. Subsequently, cells were washed three times with PBS (700 g × 5 min), and the fluorescence intensity of DHE was assayed by FACScan flow cytometer (FACS, AriaIII). For each sample 30 000 events were collected. The mean DHE fluorescence intensity was determined using FlowJo software (FlowJo 10.0.7)

### Fluorescent imaging of the mitochondrial membrane potential (Δψ_m_)

Cells cultured in 96-wells plate were washed once by PBS and incubated with 50 nM tetramethylrhodamine methylester perchlorate (TMRM) at 37 °C for 90 min before washing and mounting in Hanks’ buffered salt solution for visualization. Depolarized or inactive mitochondria have decreased membrane potential and fail to sequester TMRM. The quantification of the fluorescent intensity was done using the Image J software (Image J 1.46r, NIH, Baltimore, MD).

### Dual-luciferase Assay

The luciferase activities of truncated and mutant ANT1 promoters were examined by Dual-Luciferase^®^ Reporter Assay System (E1910; Promega, Wisconsin, USA) using GloMax 20/20 Luminometer (Promega, Wisconsin, USA) as previously described^47^. NRE1 site (5′-AAGGGGGAGCTGCGGGCCAG-3′) was mutated to NRE1 mutant (5′-AAGaaacaaCTGttGGCCAG-3′) and NRE3 (5′-CGCAGGGTCGGGGACTGCGCG-3′) was mutated to NRE3 mutant (5′-CGCAGGGTCaacaAaTGttCa-3′).

### Statistical Analysis

All the experiments were repeated at least three times. For immunoblotting, one representative picture was shown, while quantifications were calculated from at least three independent experiments. All data are shown as means ± SEM. Comparisons between 2 groups were performed using Student’s t tests and comparisons between multiple groups were performed by one-way ANOVA with Dunnett’s multiple comparison test. All the P-values had been shown in figure legends. Differences were classified as significant at P < 0.05. The original data is included in the supplementary information.

## Additional Information

**How to cite this article:** Zhang, C. *et al*. Transcription factor NF-kappa B represses ANT1 transcription and leads to mitochondrial dysfunctions. *Sci. Rep.* **7**, 44708; doi: 10.1038/srep44708 (2017).

**Publisher's note:** Springer Nature remains neutral with regard to jurisdictional claims in published maps and institutional affiliations.

## Supplementary Material

Supplementary Information

## References

[b1] Brookes P. S., Yoon Y., Robotham J. L., Anders M. W. & Sheu S. S. Calcium, ATP, and ROS: a mitochondrial love-hate triangle. Am J Physiol Cell Physiol 287, C817–833, doi: 10.1152/ajpcell.00139.2004 (2004). 15355853

[b2] Bhat A. H. . Oxidative stress, mitochondrial dysfunction and neurodegenerative diseases; a mechanistic insight. Biomed Pharmacother 74, 101–110, doi: 10.1016/j.biopha.2015.07.025 (2015). 26349970

[b3] Gautier C. A., Corti O. & Brice A. Mitochondrial dysfunctions in Parkinson’s disease. Rev Neurol (Paris) 170, 339–343, doi: 10.1016/j.neurol.2013.06.003 (2014). 24119854

[b4] Federico A. . Mitochondria, oxidative stress and neurodegeneration. J Neurol Sci 322, 254–262, doi: 10.1016/j.jns.2012.05.030 (2012). 22669122

[b5] Klingenberg M. The ADP and ATP transport in mitochondria and its carrier. Biochim Biophys Acta 1778, 1978–2021, doi: 10.1016/j.bbamem.2008.04.011 (2008). 18510943

[b6] Stepien G., Torroni A., Chung A. B., Hodge J. A. & Wallace D. C. Differential expression of adenine nucleotide translocator isoforms in mammalian tissues and during muscle cell differentiation. J Biol Chem 267, 14592–14597 (1992). 1378836

[b7] Dolce V., Scarcia P., Iacopetta D. & Palmieri F. A fourth ADP/ATP carrier isoform in man: identification, bacterial expression, functional characterization and tissue distribution. FEBS Lett 579, 633–637, doi: 10.1016/j.febslet.2004.12.034 (2005). 15670820

[b8] Dorner A., Olesch M., Giessen S., Pauschinger M. & Schultheiss H. P. Transcription of the adenine nucleotide translocase isoforms in various types of tissues in the rat. Biochim Biophys Acta 1417, 16–24 (1999). 10076031 10.1016/s0005-2736(98)00245-4

[b9] Levy S. E., Chen Y. S., Graham B. H. & Wallace D. C. Expression and sequence analysis of the mouse adenine nucleotide translocase 1 and 2 genes. Gene 254, 57–66 (2000). 10974536 10.1016/s0378-1119(00)00252-3

[b10] Gavalda-Navarro A., Villena J. A., Planavila A., Vinas O. & Mampel T. Expression of adenine nucleotide translocase (ANT) isoform genes is controlled by PGC-1alpha through different transcription factors. J Cell Physiol 229, 2126–2136, doi: 10.1002/jcp.24671 (2014). 24819348

[b11] Fiore C. . The mitochondrial ADP/ATP carrier: structural, physiological and pathological aspects. Biochimie 80, 137–150 (1998). 9587671 10.1016/s0300-9084(98)80020-5

[b12] Palmieri F. The mitochondrial transporter family (SLC25): physiological and pathological implications. Pflugers Arch 447, 689–709, doi: 10.1007/s00424-003-1099-7 (2004). 14598172

[b13] Maldonado E. N. . ATP/ADP Turnover and Import of Glycolytic ATP into Mitochondria in Cancer Cells Is Independent of the Adenine Nucleotide Translocator. J Biol Chem 291, 19642–19650, doi: 10.1074/jbc.M116.734814 (2016). 27458020 PMC5016697

[b14] Vander Heiden M. G., Chandel N. S., Schumacker P. T. & Thompson C. B. Bcl-xL prevents cell death following growth factor withdrawal by facilitating mitochondrial ATP/ADP exchange. Mol Cell 3, 159–167 (1999). 10078199 10.1016/s1097-2765(00)80307-x

[b15] Andreyev A. . The ATP/ADP-antiporter is involved in the uncoupling effect of fatty acids on mitochondria. Eur J Biochem 182, 585–592 (1989). 2546761 10.1111/j.1432-1033.1989.tb14867.x

[b16] Boudina S. & Abel E. D. Mitochondrial uncoupling: a key contributor to reduced cardiac efficiency in diabetes. Physiology (Bethesda) 21, 250–258, doi: 10.1152/physiol.00008.2006 (2006). 16868314

[b17] Azzu V., Parker N. & Brand M. D. High membrane potential promotes alkenal-induced mitochondrial uncoupling and influences adenine nucleotide translocase conformation. Biochem J 413, 323–332, doi: 10.1042/BJ20080321 (2008). 18426390 PMC2474560

[b18] Brand M. D. . The basal proton conductance of mitochondria depends on adenine nucleotide translocase content. Biochem J 392, 353–362, doi: 10.1042/BJ20050890 (2005). 16076285 PMC1316271

[b19] Bauer M. K., Schubert A., Rocks O. & Grimm S. Adenine nucleotide translocase-1, a component of the permeability transition pore, can dominantly induce apoptosis. The Journal of cell biology 147, 1493–1502 (1999). 10613907 10.1083/jcb.147.7.1493 PMC2174250

[b20] Halestrap A. P., McStay G. P. & Clarke S. J. The permeability transition pore complex: another view. Biochimie 84, 153–166 (2002). 12022946 10.1016/s0300-9084(02)01375-5

[b21] Grivennikov S. I., Greten F. R. & Karin M. Immunity, inflammation, and cancer. Cell 140, 883–899, doi: 10.1016/j.cell.2010.01.025 (2010). 20303878 PMC2866629

[b22] Ghosh S. & Karin M. Missing pieces in the NF-kappaB puzzle. Cell 109 Suppl, S81–96 (2002). 11983155 10.1016/s0092-8674(02)00703-1

[b23] Vallabhapurapu S. & Karin M. Regulation and function of NF-kappaB transcription factors in the immune system. Annu Rev Immunol 27, 693–733, doi: 10.1146/annurev.immunol.021908.132641 (2009). 19302050

[b24] Beg A. A., Finco T. S., Nantermet P. V. & Baldwin A. S. Jr. Tumor necrosis factor and interleukin-1 lead to phosphorylation and loss of I kappa B alpha: a mechanism for NF-kappa B activation. Mol Cell Biol 13, 3301–3310 (1993). 8497253 10.1128/mcb.13.6.3301-3310.1993 PMC359784

[b25] Schoonbroodt S. . Crucial role of the amino-terminal tyrosine residue 42 and the carboxyl-terminal PEST domain of I kappa B alpha in NF-kappa B activation by an oxidative stress. J Immunol 164, 4292–4300 (2000). 10754328 10.4049/jimmunol.164.8.4292

[b26] Behrens M. M., Ali S. S. & Dugan L. L. Interleukin-6 mediates the increase in NADPH-oxidase in the ketamine model of schizophrenia. J Neurosci 28, 13957–13966, doi: 10.1523/JNEUROSCI.4457-08.2008 (2008). 19091984 PMC2752712

[b27] Samavati L., Lee I., Mathes I., Lottspeich F. & Huttemann M. Tumor necrosis factor alpha inhibits oxidative phosphorylation through tyrosine phosphorylation at subunit I of cytochrome c oxidase. J Biol Chem 283, 21134–21144, doi: 10.1074/jbc.M801954200 (2008). 18534980 PMC3258931

[b28] Hunter R. L. . Inflammation induces mitochondrial dysfunction and dopaminergic neurodegeneration in the nigrostriatal system. J Neurochem 100, 1375–1386, doi: 10.1111/j.1471-4159.2006.04327.x (2007). 17254027

[b29] Cogswell P. C. . NF-kappa B and I kappa B alpha are found in the mitochondria. Evidence for regulation of mitochondrial gene expression by NF-kappa B. J Biol Chem 278, 2963–2968, doi: 10.1074/jbc.M209995200 (2003). 12433922

[b30] Guseva N. V., Taghiyev A. F., Sturm M. T., Rokhlin O. W. & Cohen M. B. Tumor necrosis factor-related apoptosis-inducing ligand-mediated activation of mitochondria-associated nuclear factor-kappaB in prostatic carcinoma cell lines. Mol Cancer Res 2, 574–584 (2004). 15498932

[b31] Pan S., Wang N., Bisetto S., Yi B. & Sheu S. S. Downregulation of adenine nucleotide translocator 1 exacerbates tumor necrosis factor-alpha-mediated cardiac inflammatory responses. Am J Physiol Heart Circ Physiol 308, H39–48, doi: 10.1152/ajpheart.00330.2014 (2015). 25380814 PMC4281676

[b32] Wang K. . MicroRNA-2861 regulates programmed necrosis in cardiomyocyte by impairing adenine nucleotide translocase 1 expression. Free radical biology & medicine 91, 58–67, doi: 10.1016/j.freeradbiomed.2015.11.031 (2016). 26654759

[b33] Baud V. & Karin M. Signal transduction by tumor necrosis factor and its relatives. Trends Cell Biol 11, 372–377 (2001). 11514191 10.1016/s0962-8924(01)02064-5

[b34] Kunsch C., Ruben S. M. & Rosen C. A. Selection of optimal kappa B/Rel DNA-binding motifs: interaction of both subunits of NF-kappa B with DNA is required for transcriptional activation. Molecular and cellular biology 12, 4412–4421 (1992). 1406630 10.1128/mcb.12.10.4412-4421.1992 PMC360365

[b35] Kokoszka J. E. . The ADP/ATP translocator is not essential for the mitochondrial permeability transition pore. Nature 427, 461–465, doi: 10.1038/nature02229 (2004). 14749836 PMC3049806

[b36] Brustovetsky N. & Klingenberg M. Mitochondrial ADP/ATP carrier can be reversibly converted into a large channel by Ca2+. Biochemistry 35, 8483–8488, doi: 10.1021/bi960833v (1996). 8679608

[b37] Lee J., Schriner S. E. & Wallace D. C. Adenine nucleotide translocator 1 deficiency increases resistance of mouse brain and neurons to excitotoxic insults. Biochim Biophys Acta 1787, 364–370, doi: 10.1016/j.bbabio.2009.01.014 (2009). 19366611 PMC3245720

[b38] Bottero V. . Ikappa b-alpha, the NF-kappa B inhibitory subunit, interacts with ANT, the mitochondrial ATP/ADP translocator. J Biol Chem 276, 21317–21324, doi: 10.1074/jbc.M005850200 (2001). 11287411

[b39] Zamora M., Merono C., Vinas O. & Mampel T. Recruitment of NF-kappaB into mitochondria is involved in adenine nucleotide translocase 1 (ANT1)-induced apoptosis. J Biol Chem 279, 38415–38423, doi: 10.1074/jbc.M404928200 (2004). 15231833

[b40] Maruszak A. & Zekanowski C. Mitochondrial dysfunction and Alzheimer’s disease. Prog Neuropsychopharmacol Biol Psychiatry 35, 320–330, doi: 10.1016/j.pnpbp.2010.07.004 (2011). 20624441

[b41] Nunomura A. . Oxidative damage to RNA in aging and neurodegenerative disorders. Neurotox Res 22, 231–248, doi: 10.1007/s12640-012-9331-x (2012). 22669748

[b42] Esposito L. A., Melov S., Panov A., Cottrell B. A. & Wallace D. C. Mitochondrial disease in mouse results in increased oxidative stress. Proc Natl Acad Sci USA 96, 4820–4825 (1999). 10220377 10.1073/pnas.96.9.4820 PMC21775

[b43] Naik E. & Dixit V. M. Mitochondrial reactive oxygen species drive proinflammatory cytokine production. J Exp Med 208, 417–420, doi: 10.1084/jem.20110367 (2011). 21357740 PMC3058577

[b44] Bulua A. C. . Mitochondrial reactive oxygen species promote production of proinflammatory cytokines and are elevated in TNFR1-associated periodic syndrome (TRAPS). J Exp Med 208, 519–533, doi: 10.1084/jem.20102049 (2011). 21282379 PMC3058571

[b45] Sun X. . Regulator of calcineurin 1 (RCAN1) facilitates neuronal apoptosis through caspase-3 activation. J Biol Chem 286, 9049–9062, doi: 10.1074/jbc.M110.177519 (2011). 21216952 PMC3059004

[b46] Zheng L., Liu H., Wang P., Song W. & Sun X. Regulator of calcineurin 1 gene transcription is regulated by nuclear factor-kappaB. Current Alzheimer research 11, 156–164 (2014). 24359503 10.2174/1567205010666131212114907

[b47] Sun X. . Distinct transcriptional regulation and function of the human BACE2 and BACE1 genes. FASEB journal: official publication of the Federation of American Societies for Experimental Biology 19, 739–749, doi: 10.1096/fj.04-3426com (2005). 15857888

[b48] Lu M. . REST regulates DYRK1A transcription in a negative feedback loop. J Biol Chem 286, 10755–10763, doi: 10.1074/jbc.M110.174540 (2011). 21252229 PMC3060526

[b49] Yang N. C., Ho W. M., Chen Y. H. & Hu M. L. A convenient one-step extraction of cellular ATP using boiling water for the luciferin-luciferase assay of ATP. Analytical biochemistry 306, 323–327 (2002). 12123672 10.1006/abio.2002.5698

[b50] Kawamata H., Starkov A. A., Manfredi G. & Chinopoulos C. A kinetic assay of mitochondrial ADP-ATP exchange rate in permeabilized cells. Analytical biochemistry 407, 52–57, doi: 10.1016/j.ab.2010.07.031 (2010). 20691655 PMC2943973

[b51] Doczi J. . Alterations in voltage-sensing of the mitochondrial permeability transition pore in ANT1-deficient cells. Sci Rep 6, 26700, doi: 10.1038/srep26700 (2016). 27221760 PMC4879635

[b52] Petronilli V. . Transient and long-lasting openings of the mitochondrial permeability transition pore can be monitored directly in intact cells by changes in mitochondrial calcein fluorescence. Biophysical journal 76, 725–734, doi: 10.1016/S0006-3495(99)77239-5 (1999). 9929477 PMC1300077

