# Supplementary material for: Transcription factor NF-kappa B represses ANT1 transcription and leads to mitochondrial dysfunctions
Source: Sci Rep. 2017 Mar 20;7:44708. doi: 10.1038/srep44708 (PMC5357787; doi:10.1038/srep44708)

Transcription factor NF-kappa B represses ANT1 transcription and leads to mitochondrial dysfunctions

Chen Zhang[1], Hui Jiang[4], Pin Wang[2], Heng Liu[2], Xiulian Sun[3†]

Figure 1. NF-κB represses ANT1 gene transcription and protein expression.

Figure 1A

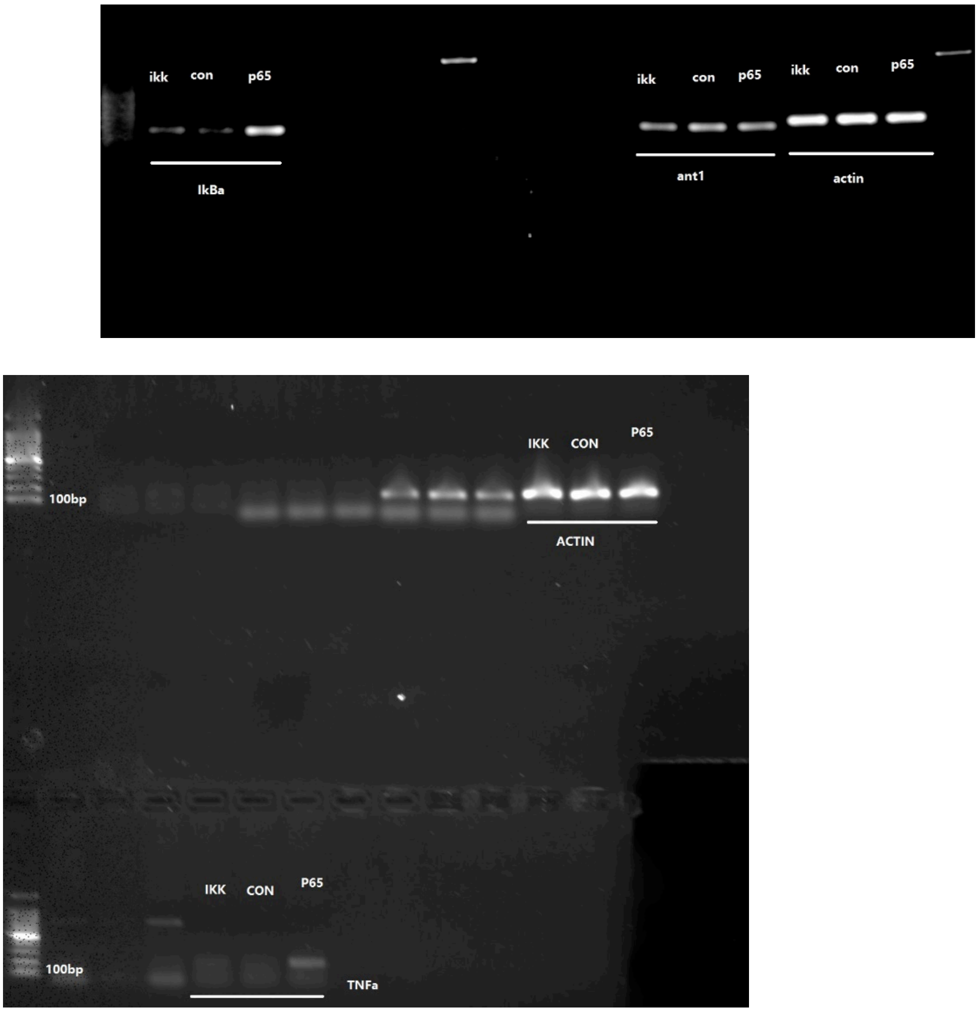

Figure 1B

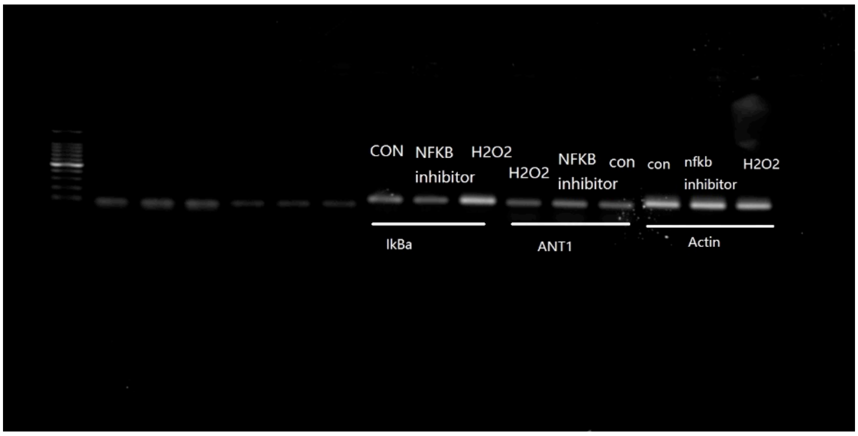

Figure 1C

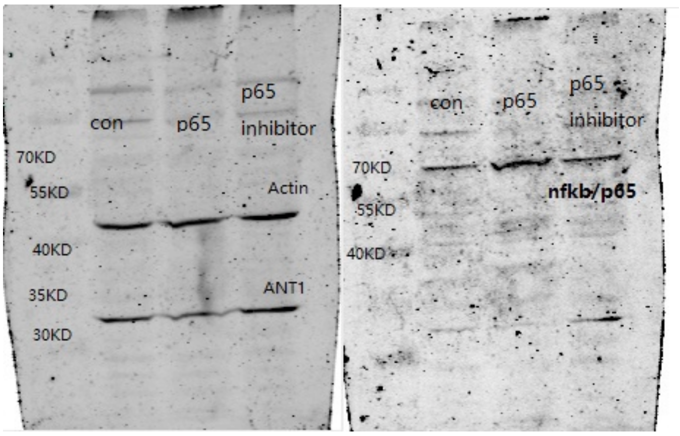

Figure 1D

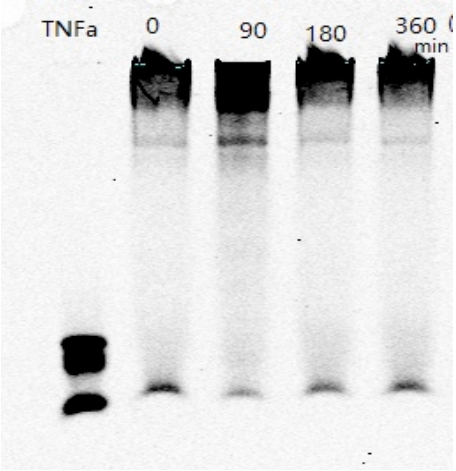

Figure 1E

minutes	mRNA of ANT1(% of con)		
0	99.832000	100.322200	99.322220
90	43.020010	48.263310	58.257160
180	80.809700	74.650890	78.035910
360	90.567910	88.058190	87.905360

minutes	p65 activity (% of con)	
0	99.410040	100.590000
90	140.458000	137.177200
180	108.207600	106.959100
360	82.437390	81.470410

Figure 1 F

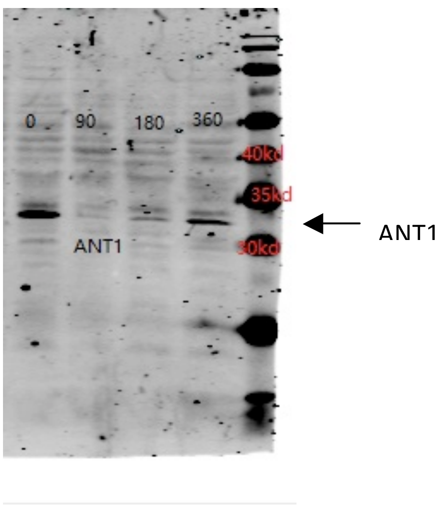
0
90
180
360
40kd
35kd
30kd
ANT1
ANT1

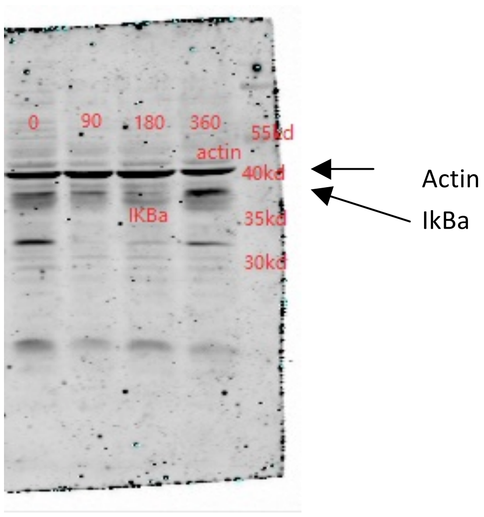
0
90
180
360
55kd
actin
40kd
IKBa
35kd
30kd
Actin
IkBa

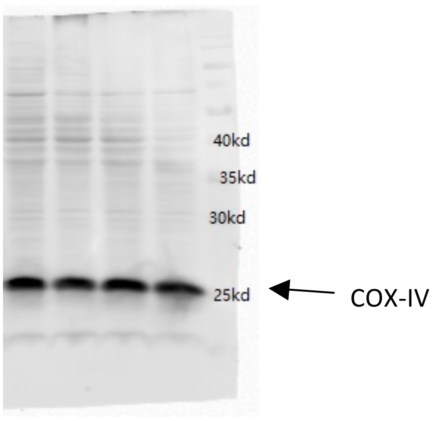
40kd
35kd
30kd
25kd
COX-IV

Figure 1G

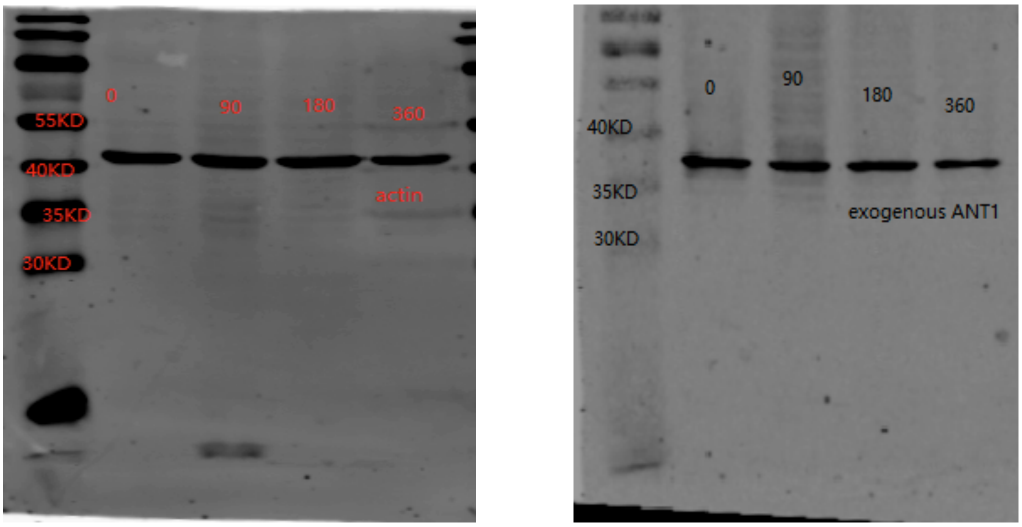
0
90
180
360
55KD
40KD
35KD
30KD
actin
0
90
180
360
40KD
35KD
30KD
exogenous ANT1

Figure 2. NF-κB negatively regulates the *ANT1* promoter.

Figure 2B

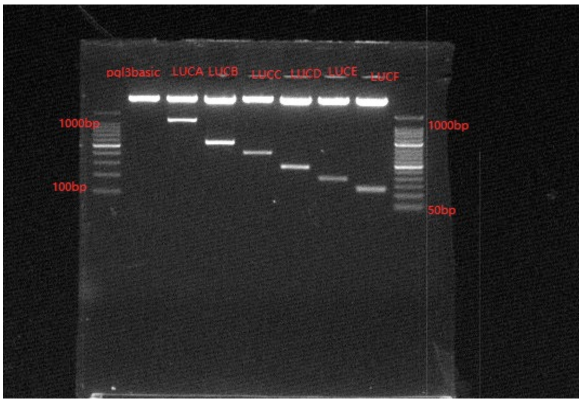

Figure 2C

	CON				NFKB			
PGL3-BASIC	0.2125667	0.1386833	0.120800	0.286300	0.2173457	0.1386833	0.120800	0.286300
ANT-LUCA	18.347270	12.760230	9.689700	16.341600	6.391556	7.822747	7.799374	5.242778
ANTLUC-B	24.215100	18.063400	11.826330		9.830753	14.578290	3.826989	
ANTLUC-C	10.498930	13.022200	10.498930	13.866830	2.265095	2.967806	2.265095	2.817522
ANTLUC-D	11.742910	8.536300	9.599000	15.089450	2.440548	2.682487	2.635335	1.989928
ANT1LUC-E	9.130867	8.858268	12.825340		3.284943	5.739098	4.821162	
ANTLUC-F	3.318300	2.078000	4.123200	3.753700	3.825549	4.530502	3.724702	3.221441
NF-KBLUC	0.145699	0.03734807			51.192490	48.512500		

Figure 2D

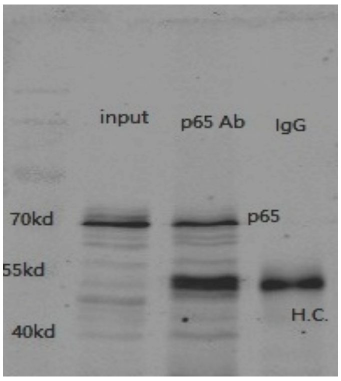

Figure 2 E

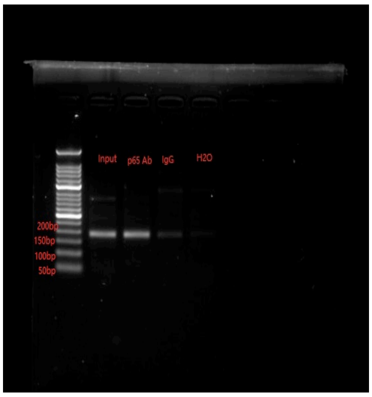

Figure 3. Identification of the functional NRE site in *ANT1* gene promoter.

Figure 3 B

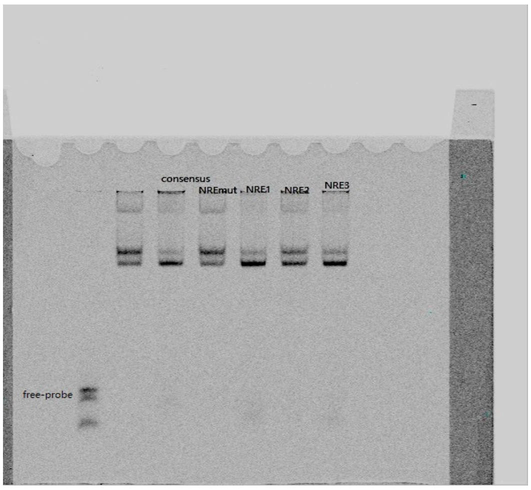

Figure 3 C

	basic			ant1luc			AntNRE1m		
con	0.098500	0.067200		2.838133	2.916400	2.519400	2.750167	2.444900	2.660300
nfkb	0.08026316	0.080198		0.8723574	0.8210855	0.9162829	0.9819408	0.9567763	0.9792106

AntNRE2m			AntNRE1 and 2 m		
1.768633	1.646600	1.823600	2.316981	2.182201	
0.7841009	0.7559211	0.8091776	2.586231	3.166899	

Figure 3D

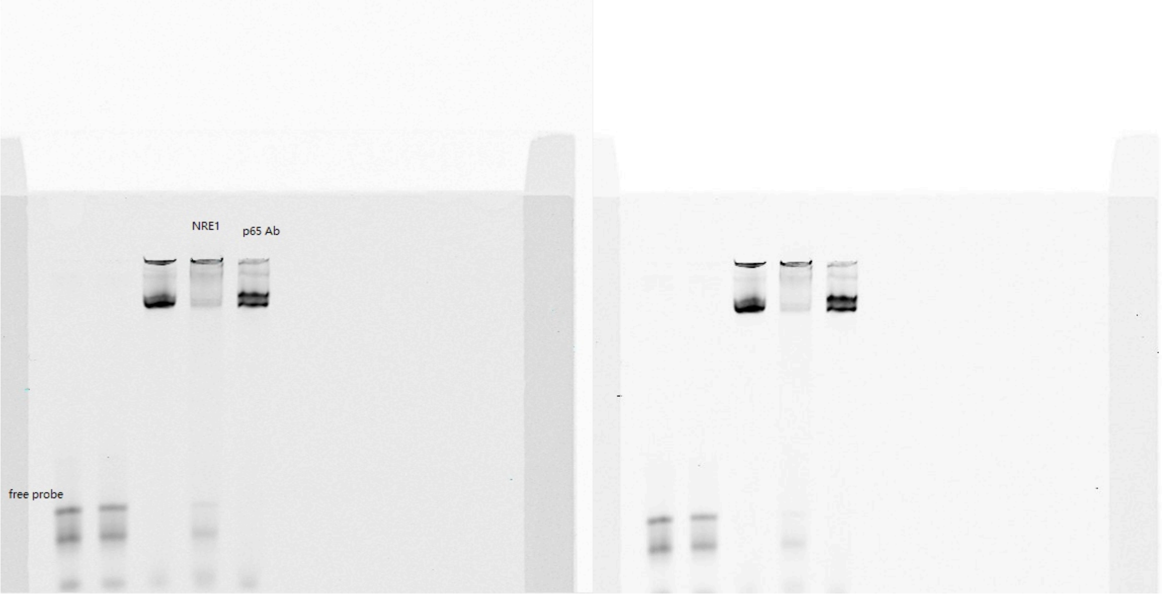

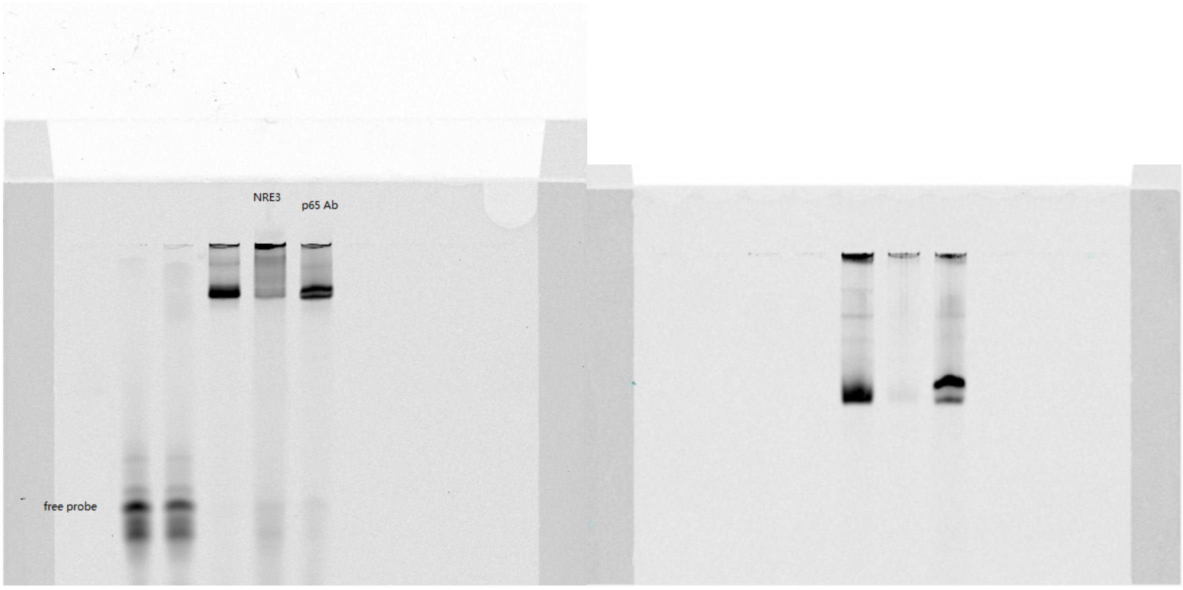
NRE3
p65 Ab
free probe

Figure 4. NF-κB impairs mitochondrial ATP/ADP exchange rate and intracellular ATP level.

Figure 4 A & 4B

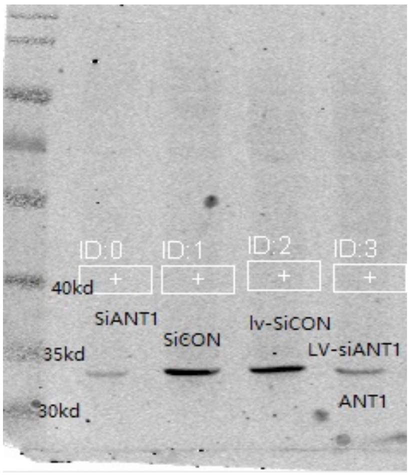

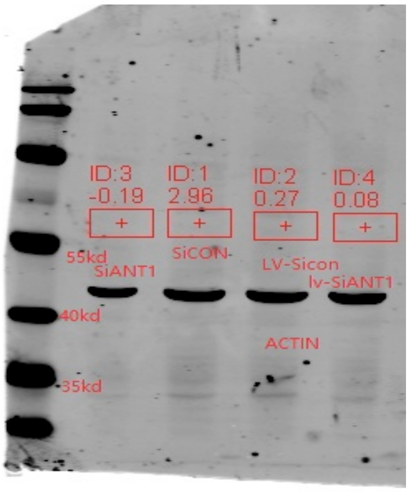

Figure 4 C

time(s)	pbs	tnfa	sicon	siant1	con	ant1
0.	0.276500	0.360000	0.312000	0.002430	0.099000	0.349000
2.	0.421000	0.383500	0.447000	0.009600	0.152000	0.456500
4.	0.493500	0.396500	0.558000	0.024100	0.164000	0.6348465
6.	0.624924	0.481000	0.636000	0.050500	0.194500	0.690824
8.	0.7988745	0.484000	0.653000	0.050500	0.408000	1.108207
10.	0.931041	0.4934854	0.725000	0.0533843	0.6784945	1.153605
12.	0.967359	0.5352125	0.871000	0.167500	0.7622925	1.214275
14.	1.042455	0.595345	0.880000	0.193000	0.7824445	1.264018
16.	1.043991	0.6002395	0.885000	0.276500	0.7916425	1.425709
18.	1.057489	0.670940	0.943000	0.296500	0.8223915	1.435380
20.	1.057812	0.683432	1.060000	0.305500	0.898827	1.438051
22.	1.113920	0.725688	1.240000	0.3475032	1.029155	1.552686
24.	1.164991	0.7894765	1.290000	0.390000	1.062766	1.560166
26.	1.184054	0.801534	1.350000	0.404500	1.112118	1.698272
28.	1.214275	0.810846	1.390000	0.443500	1.119665	1.698504
30.	1.273484	0.8202825	1.440000	0.446500	1.161162	1.740010
32.	1.310965	0.822494	1.440000	0.457500	1.195421	1.799705
34.	1.339325	0.832182	1.490000	0.478500	1.227142	1.909989
36.	1.351665	0.879223	1.500000	0.485500	1.390000	1.964965
38.	1.509447	0.889841	1.520000	0.497000	1.445000	2.062413
40.	1.560866	0.8981875	1.530000	0.510279	1.545000	2.105196
42.	1.576590	0.9435815	1.530000	0.517250	1.575000	2.308598
44.	1.617604	0.9527325	1.560000	0.5494745	1.580000	2.419562
46.	1.661979	1.005198	1.640000	0.584816	1.645000	2.453634
48.	1.749925	1.083135	1.690000	0.596498	1.855000	2.482224
50.	1.819980	1.136754	1.710000	0.624416	1.880000	2.554734
52.	1.850301	1.177052	1.750000	0.6492525	1.885000	2.632395
54.	1.858539	1.189593	1.810000	0.675436	1.955000	2.846791

56.	1.864714	1.192062	1.850000	0.700719	2.015000	2.896856
58.	1.877512	1.224938	1.860000	0.709831	2.090000	2.983932
60.	1.920546	1.251119	1.900000	0.737515	2.126012	3.038430
62.	2.106021	1.261891	1.950000	0.7387475	2.142285	3.078340
64.	2.170628	1.314628	1.960000	0.765911	2.219531	3.092393
66.	2.279375	1.321464	2.010000	0.7719825	2.248430	3.124391
68.	2.529195	1.352468	2.020000	0.7779745	2.326253	3.132170
70.	2.579140	1.367162	2.030000	0.780048	2.511553	3.242995
72.	2.730661	1.400931	2.050000	0.835455	2.656154	3.347069
74.	2.845364	1.409221	2.060000	0.8420305	2.706593	3.351426
76.	2.965110	1.449785	2.060000	0.844782	2.765800	3.402659
78.	3.066736	1.452145	2.070000	0.8485265	2.770497	3.407072
80.	3.097731	1.535499	2.080000	0.8534915	2.844644	3.567547
82.	3.190224	1.547534	2.090000	0.905708	2.922116	3.603434
84.	3.410173	1.550248	2.090000	1.049649	2.967868	3.704027
86.	3.607660	1.564549	2.160000	1.108315	3.019881	3.731255
88.	3.614541	1.589664	2.170000	1.181201	3.026556	3.753982
90.	3.703892	1.596981	2.230000	1.212257	3.215570	4.073921
92.	3.783902	1.629093	2.240000	1.256297	3.337086	4.177380
94.	3.877075	1.630196	2.270000	1.266896	3.360915	4.701469
96.	3.892448	1.641292	2.340000	1.337473	3.435154	4.702245
98.	4.055562	1.650542	2.340000	1.413847	3.483841	4.718666
100.	4.120077	1.662110	2.350000	1.477476	3.487393	4.886819
102.	4.246793	1.680208	2.370000	1.493302	3.621829	4.915195
104.	4.316838	1.705057	2.430000	1.566288	3.845033	4.927385
106.	4.329782	1.733696	2.660000	1.566795	3.892255	5.127945
108.	4.560615	1.743067	2.830000	1.600829	3.899092	5.140245
110.	4.620308	1.860229	2.840000	1.610373	3.931779	5.144575
112.	4.629973	1.862642	2.850000	1.646320	3.941417	5.203135
114.	4.853141	1.883136	2.940000	1.656697	4.003627	5.489915
116.	4.901331	1.991025	2.980000	1.669585	4.267840	5.500335
118.	4.972956	2.075603	3.000000	1.702222	4.403863	5.539590
120.	5.105695	2.101312	3.250000	1.790121	4.410164	5.809720
122.	5.159095	2.106591	3.260000	1.799324	4.548236	5.910280
124.	5.300880	2.167130	3.470000	1.800037	4.573589	5.923830
126.	5.320730	2.252743	3.510000	1.828743	4.614141	5.989800
128.	5.350830	2.300161	3.550000	1.832800	4.749402	5.991620
130.	5.499955	2.391170	3.660000	1.842756	4.826302	6.391660
132.	5.539905	2.479080	3.680000	1.845243	5.151925	6.397550
134.	5.654155	2.520478	3.700000	1.862718	5.172985	6.483580
136.	5.702615	2.529816	3.990000	1.870242	5.186985	6.640440
138.	5.838165	2.545885	4.180000	1.915580	5.234555	6.662475
140.	5.905760	2.611191	4.250000	1.917135	5.528470	6.851535
142.	6.074785	2.738298	4.390000	1.937206	5.564385	7.127105
144.	6.152595	2.760507	4.510000	1.937983	5.631305	7.201145

146.	6.197130	2.786197	4.580000	1.946242	5.805320	7.451165
148.	6.204580	2.792769	4.810000	1.951182	5.839400	7.507995
150.	6.264830	2.976338	4.890000	1.966272	5.866260	7.766135
152.	6.450430	3.038193	4.940000	1.972613	5.870475	7.908175
154.	6.587165	3.051906	5.200000	2.007231	6.060920	7.948610
156.	6.700975	3.056530	5.480000	2.024579	6.130845	8.031280
158.	6.731260	3.070339	5.610000	2.041275	6.245990	8.401640
160.	6.769040	3.108697	5.630000	2.080203	6.290870	8.645835
162.	6.879565	3.117079	5.700000	2.084398	6.317715	9.023235
164.	7.100055	3.124515	5.720000	2.092434	6.425275	9.182860
166.	7.149800	3.138598	5.820000	2.125650	6.476155	9.242085
168.	7.160710	3.219981	6.150000	2.132897	6.506930	9.473830
170.	7.315810	3.227811	6.400000	2.141533	6.549020	9.723635
172.	7.410960	3.278339	6.490000	2.174984	6.701500	10.896470
174.	7.461550	3.340383	6.630000	2.223389	6.873920	11.004650
176.	7.486685	3.429838	6.680000	2.239305	6.875040	11.533840
178.	7.669225	3.494483	6.710000	2.241656	6.985175	11.771970
180.	7.846155	3.567809	6.880000	2.277431	7.029530	12.137370
182.	8.070685	3.595835	6.960000	2.277845	7.084225	12.242440
184.	8.176835	3.703107	7.230000	2.308507	7.121770	12.667310
186.	8.192675	3.711419	7.700000	2.312500	7.125155	12.691820
188.	8.377005	3.758111	7.710000	2.312982	7.146175	13.068380
190.	8.464540	3.817218	7.740000	2.315527	7.193290	13.370540
192.	8.525490	3.899935	7.760000	2.324193	7.262390	13.710060
194.	8.538310	3.953311	7.830000	2.402988	7.268670	13.758920
196.	8.956030	4.020369	8.040000	2.547287	7.291845	14.022340
198.	8.963525	4.061947	8.100000	2.854898	7.339750	14.949600

Exchange rate (times of con)

pbs	tnfa	sicon	siant1	con	ant1
1.005917	0.7647059	1.0000	0.437500	0.9797918	1.375000
0.9467456	0.750000	1.0625	0.4117647	1.041029	2.058824
1.053254	0.7752809	1.0000	0.562500	0.9797918	1.587500

Figure 4D

ATP level in T98G(nM/10^4 cells)

con	siant1	pbs	tnfa
515.5289	191.2706	400.5951	237.2905
396.9066	200.646	347.3538	216.3816
390.887	156.5128	292.3562	166.1956
274.3893	148.6237	264.9036	124.9429
394.4279		326.3022	

ATP level in T98G(times of con)

con	siant1	pbs	tnfa
1.308449	0.371018	1.228819	0.592345
1.007377	0.505524	1.065502	0.622943
0.992099	0.400404	0.896798	0.56847
0.696419	0.541653	0.812588	0.471654

Figure 4 E

ATP level in neuron (nM/10^4 cells)

lv-sicon	lv-siant1	pbs	tnfa
77.26735	44.3427	103.4948	78.79586
64.78874	39.44631	93.38913	69.27648
67.48053	27.03529	86.43686	43.90684
56.93962	23.99309	81.61415	40.94602
67.48053	27.03529	76.67759	39.00787

ATP level in neuron (times of con)

lv-sicon	lv-siant1	pbs	tnfa
1.15687	0.573887	1.176077	0.761351
0.970037	0.608845	1.06124	0.741805
1.010339	0.400638	0.982237	0.507964
0.852517	0.421378	0.927433	0.501703
1.010339	0.400638	0.871336	0.508726

Figure 4 F

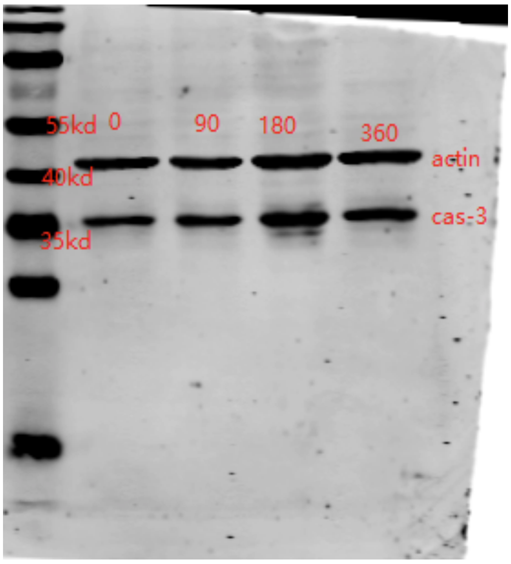

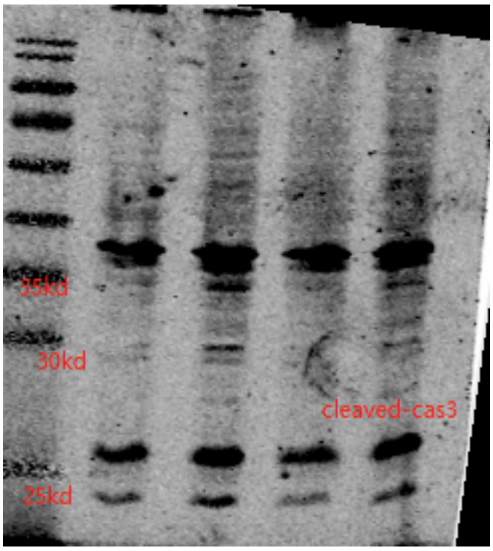

Figure 5. NF-κB decreased Ca^{2+}-induced mPTP opening, increased $\Delta\psi_m$ and ROS.

Figure 5A

mPTP opening of T98G(% of con)

	pbs			
0um ionomycin	99.374925	99.4282458	99.5793213	100.91678
5um ionomycin	68.7857181	69.2188385	69.0753028	70.51375
5um ionomycin+bka	90.4900811	89.4649878	91.8190711	89.22635
5um ionomycin+catr	57.8558646	57.376259	55.0638445	57.177
	tnf			
0um ionomycin	99.6298772	98.9076505	99.8144955	100.83162
5um ionomycin	79.1744633	79.753279	78.1536137	77.60367
5um ionomycin+bka	89.8088362	90.1559449	90.9570167	91.99262
5um ionomycin+catr	68.745843	63.7093395	68.4697549	61.64629
	sicon			
0um ionomycin	99.8461202	99.4303825	99.2638251	101.09519
5um ionomycin	72.5472638	73.7458139	71.0546709	71.47954
5um ionomycin+bka	88.4357316	86.0143169	89.1396063	88.1068
5um ionomycin+catr	56.4607801	57.7953876	57.0481819	54.23096
	siant1			
0um ionomycin	98.855949	98.2012748	100.5161473	101.2133
5um ionomycin	79.2252997	80.9036831	81.297288	80.2874
5um ionomycin+bka	83.3282665	82.021836	84.3977136	81.1082
5um ionomycin+catr	71.7403765	75.3309259	69.4848702	71.25154

Figure 5 B

mPTP opening of neuron (% of con)

	pbs			
0um ionomycin	99.29078	100.7092		
5um ionomycin	71.14286	69.49296	68.98212	69.63169
5um ionomycin+bka	78.7156	80.42042		
5um ionomycin+catr	52.20317	54.89081	54.57143	56.26099
	tnfa			
0um ionomycin	98.95161	101.2903		
5um ionomycin	72.27044	78.23129	73.22721	72.58993
5um ionomycin+bka	80.77947	82.75049		
5um ionomycin+catr	59.63734	61.93481	58.48303	57.45448
	sicon			
0um ionomycin	99.38462	100.6923		
5um ionomycin	72.53844	70.75456	72.61514	69.80287
5um ionomycin+bka	85.29412	84.41558		
5um ionomycin+catr	52.99396	54.77292	52.46359	52.36025
	siant1			
0um ionomycin	100.0749	99.92		
5um ionomycin	78.92562	77.3064	76.50367	74.8328
5um ionomycin+bka	90.41198	89.74551		
5um ionomycin+catr	68.98212	69.63169	66.27878	65.68751

Fgure 5 C

time(s)	sicon+catr	siant+catr	sicon	siant1	sicon+bka	siant+bka
0	1	1	1	1	1	1
3	0.999247409	0.998673	0.997418849	0.995114	0.99878	0.999001
6	0.995692825	0.998638	0.984044324	0.994699	0.997526	0.998653
9	0.994476912	0.998552	0.981239838	0.992966	0.996046	0.997411
12	0.986183701	0.996755	0.970533409	0.992812	0.995677	0.996241
15	0.984502015	0.996748	0.96745619	0.989433	0.995515	0.994451
18	0.975757739	0.994936	0.961293394	0.985558	0.995215	0.991909
21	0.973232759	0.992775	0.959879574	0.981714	0.99401	0.990709
24	0.962539591	0.992248	0.954816218	0.977209	0.993607	0.99047
27	0.961277101	0.992212	0.944805122	0.972989	0.992815	0.990024
30	0.949860758	0.989887	0.944771835	0.968437	0.991925	0.98994
33	0.943707651	0.988789	0.934727452	0.963682	0.99159	0.989926
36	0.929947735	0.987056	0.933040899	0.959892	0.9892	0.989724
39	0.923333758	0.984745	0.930917914	0.955702	0.988197	0.989659
42	0.908014728	0.982884	0.914699798	0.952343	0.987292	0.989413
45	0.894112629	0.979617	0.91437405	0.94951	0.986726	0.988981
48	0.87258656	0.979353	0.903461499	0.947169	0.986662	0.987185
51	0.836677911	0.970161	0.895026346	0.945047	0.986028	0.985659
54	0.803426619	0.960668	0.890311575	0.943771	0.985836	0.985137
57	0.772501741	0.949457	0.88951435	0.942337	0.98378	0.984972
60	0.737610437	0.940378	0.888597113	0.940857	0.982984	0.984952
63	0.715491121	0.93008	0.887968477	0.939823	0.98292	0.984646
66	0.693715006	0.923383	0.887431279	0.938893	0.981848	0.984579
69	0.686115551	0.916529	0.886785498	0.937025	0.980185	0.984548
72	0.675581726	0.916208	0.88668263	0.935587	0.980126	0.982113
75	0.66757043	0.915816	0.875336777	0.934015	0.979698	0.979653
78	0.662991145	0.913727	0.874182373	0.931635	0.979246	0.979395
81	0.662689619	0.912821	0.874048074	0.930171	0.978371	0.977667
84	0.660377914	0.908934	0.873533735	0.927353	0.977908	0.977611
87	0.66013277	0.906595	0.863508018	0.925058	0.977662	0.976749
90	0.659272315	0.902744	0.863010824	0.922125	0.975951	0.974679
93	0.65846089	0.902629	0.862476483	0.919465	0.97301	0.972482
96	0.657497475	0.897737	0.862099302	0.916694	0.972479	0.96975
99	0.65721556	0.897209	0.860653439	0.914288	0.970831	0.969708
102	0.657051313	0.894478	0.85804077	0.911501	0.970605	0.968185
105	0.657031702	0.890655	0.850173389	0.908994	0.966936	0.966109
108	0.657002285	0.885292	0.850090524	0.906715	0.966041	0.964908
111	0.656901776	0.883723	0.850201107	0.904366	0.964935	0.964526
114	0.656838038	0.882311	0.849162209	0.902291	0.963184	0.964338
117	0.656708112	0.874787	0.848357781	0.901076	0.9622	0.963244

120	0.656695855	0.870536	0.848253362	0.899058	0.958994	0.962894
123	0.656436003	0.870287	0.845536106	0.898039	0.95859	0.961623
126	0.656372265	0.868233	0.843355182	0.897144	0.958472	0.961244
129	0.656357557	0.864175	0.840616568	0.89586	0.958261	0.960164
132	0.656266854	0.863733	0.837512489	0.895172	0.95768	0.959538
135	0.656215373	0.860288	0.835519043	0.894414	0.957572	0.959241
138	0.656171248	0.854497	0.834045318	0.893649	0.957007	0.958994
141	0.656082996	0.853235	0.833703584	0.892873	0.956456	0.958921
144	0.656078093	0.849918	0.832334277	0.892016	0.955576	0.958739
147	0.655879527	0.843978	0.831102613	0.891554	0.954803	0.957502
150	0.655823144	0.835619	0.830226921	0.890686	0.954464	0.957339
153	0.655808435	0.830712	0.828658269	0.889152	0.953844	0.955995
156	0.655805983	0.824258	0.826311224	0.888418	0.953171	0.954548
159	0.655791275	0.823317	0.825594533	0.886692	0.952772	0.952514
162	0.655448074	0.822104	0.824692737	0.885558	0.952074	0.952348
165	0.655372079	0.821263	0.824472034	0.884105	0.951676	0.951683
168	0.655239702	0.819794	0.824412705	0.882356	0.95113	0.951417
171	0.65523725	0.817297	0.82352752	0.880219	0.950589	0.951254
174	0.65522009	0.816221	0.823425475	0.878012	0.949723	0.951131
177	0.655139193	0.815999	0.823325803	0.876425	0.949399	0.950937
180	0.655041135	0.814316	0.823003054	0.874518	0.948941	0.950556
183	0.654876889	0.812676	0.822015824	0.872219	0.948808	0.948303
186	0.654644002	0.812134	0.819936945	0.870378	0.948061	0.946996
189	0.654271384	0.811877	0.817675334	0.868103	0.947933	0.945658
192	0.65353105	0.811285	0.816033115	0.865792	0.947903	0.945273
195	0.652996637	0.810758	0.813951863	0.863617	0.947643	0.94478
198	0.652351909	0.810137	0.812452033	0.861456	0.947618	0.944763
201	0.651888587	0.804681	0.810470453	0.859753	0.947559	0.944653
204	0.65153558	0.804603	0.808977742	0.85817	0.947008	0.943649
207	0.651064904	0.797535	0.808004751	0.856651	0.946649	0.943315
210	0.651055098	0.789533	0.805444123	0.854537	0.946467	0.941548
213	0.650986458	0.779684	0.803963278	0.852973	0.946295	0.941234
216	0.650312313	0.770449	0.802356656	0.852473	0.946162	0.940465
219	0.649922535	0.769529	0.800028596	0.851032	0.945848	0.939693
222	0.649564625	0.768865	0.799117307	0.850094	0.945813	0.939281
225	0.649508242	0.767525	0.798602334	0.84939	0.945803	0.938428
228	0.649307224	0.766455	0.798398243	0.848137	0.945641	0.938193
231	0.649304772	0.766241	0.798372138	0.847414	0.945248	0.937617
234	0.648804679	0.765478	0.797954464	0.846515	0.944859	0.937511
237	0.648451672	0.764836	0.796051198	0.845992	0.944574	0.93642
240	0.64766231	0.76403	0.795512493	0.845296	0.944471	0.934588
243	0.647125445	0.762989	0.795303656	0.8452	0.944471	0.931723
246	0.64692933	0.76142	0.793476331	0.844601	0.944451	0.930164
249	0.646184093	0.760707	0.791770037	0.844235	0.944235	0.93004
252	0.645956109	0.760543	0.790984525	0.843897	0.944171	0.929656
255	0.645929144	0.760136	0.790255968	0.842821	0.943881	0.929476
258	0.645840892	0.757925	0.788843944	0.841718	0.943512	0.929403

261	0.645835989	0.757726	0.786477914	0.841641	0.943084	0.929288
264	0.645823732	0.756485	0.783328745	0.840595	0.942828	0.928893
267	0.645642326	0.755836	0.782512382	0.840126	0.942749	0.92848
270	0.645554074	0.755172	0.781843154	0.839327	0.9427	0.927877
273	0.645306479	0.753475	0.7813353	0.838112	0.942464	0.92783
276	0.645196164	0.751371	0.780791849	0.837262	0.942041	0.927426
279	0.644852963	0.751364	0.775663473	0.836175	0.941825	0.927198
282	0.644772065	0.751236	0.773349653	0.835794	0.94181	0.926797
285	0.644757357	0.751171	0.771785747	0.835052	0.941702	0.926632
288	0.644526922	0.749988	0.770501874	0.834764	0.941535	0.926573
291	0.644495053	0.749695	0.76904476	0.833718	0.941303	0.926407
294	0.644345515	0.749467	0.767727662	0.832761	0.941205	0.926371
297	0.644213138	0.748283	0.765845755	0.832012	0.941161	0.926354
300	0.64399496	0.746907	0.765511141	0.830958	0.941141	0.926306
303	0.64390916	0.746792	0.763956728	0.82999	0.941122	0.926259
306	0.643842971	0.746379	0.76370992	0.828825	0.941107	0.925294
309	0.643570861	0.745673	0.763501083	0.827618	0.941028	0.92489
312	0.64356841	0.744838	0.763493964	0.826499	0.941023	0.924834
315	0.643404164	0.744303	0.763128499	0.825327	0.940748	0.924601
318	0.643190888	0.744254	0.760116973	0.825216	0.940605	0.924553
321	0.642185799	0.742078	0.759986449	0.825096	0.940403	0.924222
324	0.641950461	0.741921	0.759276878	0.825062	0.940394	0.923992
327	0.641945558	0.741444	0.758284901	0.824897	0.940349	0.923933
330	0.64193085	0.741116	0.758270662	0.824785	0.9403	0.923647
333	0.641859758	0.741016	0.757686868	0.824589	0.940212	0.923624
336	0.641702866	0.739875	0.757582449	0.824551	0.940069	0.922297
339	0.6416759	0.736573	0.756820668	0.824497	0.940059	0.922267
342	0.64157294	0.733863	0.753294168	0.824431	0.940025	0.922236
345	0.641536169	0.732543	0.751552277	0.824343	0.939489	0.921933
348	0.641472431	0.732486	0.75133632	0.824128	0.939346	0.921708
351	0.641403791	0.732294	0.750757272	0.824112	0.939081	0.921417
354	0.641376825	0.731858	0.750102283	0.823563	0.939012	0.921043
357	0.64128367	0.730311	0.749936162	0.823397	0.938899	0.921043
360	0.641099813	0.728521	0.749929043	0.823236	0.938874	0.921041
363	0.640825252	0.726082	0.749736818	0.823109	0.938741	0.920971
366	0.640700228	0.722452	0.749698847	0.823032	0.938525	0.920645
369	0.640673263	0.721853	0.749349994	0.822536	0.938461	0.920432
372	0.640643845	0.721603	0.748906216	0.822479	0.938294	0.920311
375	0.640619331	0.721574	0.748533631	0.822452	0.937974	0.920104
378	0.640403605	0.720854	0.748441078	0.82196	0.937025	0.919916
381	0.640141301	0.720405	0.748187151	0.821856	0.936346	0.91975
384	0.639503927	0.720341	0.747850164	0.82166	0.93614	0.919573
387	0.639334778	0.72007	0.746288632	0.821579	0.935884	0.919383
390	0.639315167	0.719763	0.745078326	0.821498	0.935608	0.918255
393	0.639192595	0.719756	0.744988146	0.821425	0.934389	0.917186
396	0.63899648	0.718978	0.744859996	0.821249	0.933922	0.915668
399	0.638766045	0.71895	0.74477219	0.821118	0.933922	0.912661

402	0.638653279	0.718836	0.743896497	0.82091	0.933848	0.911848
405	0.638572381	0.718593	0.743882259	0.820891	0.933508	0.911435
408	0.638547867	0.718572	0.74386802	0.820472	0.933317	0.911284
411	0.638091899	0.718429	0.74364969	0.820407	0.93314	0.911258
414	0.63790559	0.718244	0.743305583	0.820203	0.932402	0.911236
417	0.637675155	0.718222	0.742740774	0.820176	0.932215	0.911216
420	0.637165256	0.718215	0.742050187	0.820153	0.932082	0.91063
423	0.63667742	0.717588	0.740329654	0.820042	0.931123	0.910549
426	0.636464145	0.716304	0.740156414	0.81983	0.930744	0.910439
429	0.635976309	0.715883	0.740142175	0.819665	0.930454	0.910229
432	0.635714006	0.715092	0.739809934	0.819619	0.930105	0.91019
435	0.635505633	0.715027	0.739800442	0.819388	0.930076	0.909553
438	0.635417382	0.713444	0.739743486	0.81925	0.929933	0.909539
441	0.635392867	0.71311	0.73965568	0.819223	0.929869	0.909168
444	0.635250684	0.712783	0.739465828	0.819207	0.92981	0.908433
447	0.63522617	0.711183	0.739451589	0.818923	0.929776	0.908349
450	0.635218815	0.709978	0.739425484	0.818908	0.929648	0.908229
453	0.635174689	0.706305	0.739214274	0.818596	0.929417	0.907735
456	0.635167335	0.705146	0.739192916	0.818489	0.929328	0.907432
459	0.635103598	0.705011	0.739102736	0.81845	0.929323	0.907362
462	0.635088889	0.703965	0.739088497	0.818408	0.929215	0.907219
465	0.635071729	0.702029	0.738704047	0.818204	0.928999	0.907168
468	0.634946706	0.701436	0.738476225	0.818177	0.928782	0.907123
471	0.63490258	0.700224	0.738381299	0.817877	0.928581	0.907017
474	0.634860905	0.699755	0.736734333	0.817731	0.928551	0.906952
477	0.634738334	0.698765	0.73596306	0.817689	0.927361	0.906565
480	0.634730979	0.698688	0.735953567	0.817482	0.927263	0.906203
483	0.634718722	0.698464	0.735861014	0.817297	0.926835	0.905976
486	0.634716271	0.698104	0.735524027	0.817243	0.925767	0.905743
489	0.634684402	0.697938	0.734247273	0.817189	0.925669	0.905611
492	0.634623116	0.697751	0.734149974	0.817143	0.925089	0.904946
495	0.634620665	0.697331	0.73398148	0.816905	0.92502	0.904879
498	0.634539767	0.69722	0.733922151	0.816886	0.92499	0.904677
501	0.634534864	0.696518	0.733879435	0.816736	0.922561	0.904383
504	0.634512801	0.696278	0.733127146	0.816578	0.922408	0.90397
507	0.634480933	0.695986	0.733096295	0.816544	0.921243	0.903883
510	0.634478481	0.695899	0.733044086	0.816405	0.920717	0.903858
513	0.634439258	0.695806	0.732794905	0.816401	0.918184	0.902865
516	0.634422098	0.695626	0.732728457	0.816148	0.918017	0.902211
519	0.634412292	0.695542	0.732683367	0.815802	0.917879	0.900421
522	0.634409841	0.695481	0.732683367	0.815702	0.916045	0.900082
525	0.634402487	0.695379	0.732626412	0.81556	0.91603	0.898991
528	0.63439023	0.695176	0.732583695	0.815452	0.915061	0.898205
531	0.634358361	0.694992	0.732462664	0.815406	0.914712	0.898169
534	0.634252949	0.694966	0.732424694	0.815306	0.912204	0.897838
537	0.634235789	0.694802	0.732334514	0.814921	0.910399	0.89779
540	0.634130377	0.69473	0.731767332	0.814564	0.908077	0.896769

543	0.633948971	0.694193	0.731693764	0.814549	0.907797	0.896174
546	0.633944068	0.694038	0.730594996	0.814522	0.907654	0.896126
549	0.633907296	0.693751	0.727951308	0.81423	0.907197	0.896124
552	0.633890136	0.693489	0.72766653	0.813799	0.90703	0.896023
555	0.633887685	0.693372	0.726698285	0.813791	0.906961	0.89554
558	0.633872976	0.692771	0.725867683	0.81363	0.90551	0.895397
561	0.633831302	0.692572	0.72452448	0.813292	0.905259	0.89529
564	0.63381169	0.692076	0.724211225	0.813138	0.905048	0.895223
567	0.63381169	0.691982	0.723762699	0.8129	0.904679	0.895206
570	0.633784725	0.691666	0.72344707	0.81283	0.9043	0.895203
573	0.633762662	0.691456	0.723404354	0.81275	0.903405	0.895083
576	0.633728342	0.691283	0.723397234	0.812696	0.902825	0.894825
579	0.633676861	0.691199	0.723378249	0.812488	0.902613	0.894802
582	0.633664604	0.691089	0.723299935	0.812408	0.902023	0.894701
585	0.633659701	0.690924	0.723131442	0.812319	0.900036	0.893949
588	0.633603318	0.690544	0.72297956	0.812227	0.899033	0.893144
591	0.633603318	0.690433	0.722281854	0.81215	0.89714	0.892614
594	0.633544484	0.689994	0.722269988	0.811954	0.89652	0.892185
597	0.633441523	0.688175	0.721137996	0.811881	0.895256	0.892151

Fgure 5 D

TIME(S)	PBS+CATR	TNFa+CATR	PBS	TNFa	PBS+BKA	TNFa+BKA
0	1	1	0.996792	1	1	1
3	0.9939262	0.997419	0.995245	0.999793	0.998903	0.999783
6	0.9820486	0.99501	0.995218	0.99891	0.998588	0.999371
9	0.9805734	0.984144	0.989173	0.997987	0.99804	0.999252
12	0.9775365	0.983108	0.98368	0.996423	0.997664	0.999252
15	0.9696515	0.982564	0.977955	0.994469	0.99726	0.998929
18	0.9611918	0.979904	0.968507	0.991539	0.99691	0.998916
21	0.9588578	0.973199	0.96509	0.990458	0.996388	0.998649
24	0.9523303	0.969477	0.964341	0.98849	0.996054	0.998613
27	0.9428594	0.967692	0.962764	0.984691	0.994254	0.998587
30	0.9331219	0.967554	0.95964	0.980014	0.993856	0.998445
33	0.929846	0.967343	0.959586	0.979659	0.992496	0.998432
36	0.920434	0.966825	0.957229	0.973728	0.991267	0.998297
39	0.9176394	0.965432	0.956402	0.968385	0.990988	0.998218
42	0.9124036	0.962957	0.954054	0.968306	0.990937	0.998136
45	0.9083763	0.962207	0.953245	0.962687	0.99086	0.998024
48	0.9065029	0.961818	0.952794	0.957196	0.990533	0.997786
51	0.8937257	0.957704	0.952343	0.953269	0.98941	0.997444
54	0.8936426	0.956966	0.951623	0.951202	0.988961	0.996768
57	0.8934729	0.954022	0.951513	0.945291	0.98847	0.996403
60	0.8898785	0.950238	0.951489	0.944295	0.98787	0.996235
63	0.8874268	0.942161	0.948833	0.942168	0.987411	0.995846
66	0.8851447	0.931863	0.948726	0.939825	0.987218	0.995253

69	0.8829327	0.930085	0.94855	0.939613	0.987016	0.995042
72	0.882436	0.927123	0.945276	0.93945	0.98683	0.994957
75	0.8767043	0.924578	0.94461	0.939361	0.986112	0.994117
78	0.8709055	0.921165	0.943965	0.93902	0.984841	0.99273
81	0.8510176	0.912944	0.943675	0.938942	0.984722	0.992255
84	0.8450718	0.909473	0.942388	0.938493	0.984719	0.992213
87	0.8439152	0.905552	0.939757	0.938182	0.984572	0.990608
90	0.8424366	0.898786	0.939114	0.938172	0.984472	0.989413
93	0.8413943	0.898019	0.938494	0.937905	0.98443	0.988925
96	0.8382465	0.895638	0.938212	0.935207	0.983831	0.98826
99	0.8362312	0.894543	0.933347	0.934699	0.98343	0.98822
102	0.8356944	0.891553	0.928665	0.932705	0.983057	0.987808
105	0.8353447	0.89141	0.922963	0.929943	0.982708	0.987449
108	0.8347387	0.891363	0.918187	0.929331	0.982528	0.987311
111	0.8346452	0.890633	0.913353	0.92753	0.982178	0.987084
114	0.8344963	0.890342	0.90851	0.923415	0.981973	0.986632
117	0.8344824	0.889834	0.904154	0.921126	0.981954	0.986497
120	0.8332254	0.889823	0.899186	0.918122	0.981906	0.986099
123	0.8330938	0.889262	0.895036	0.913178	0.981854	0.98516
126	0.8329657	0.888961	0.891383	0.909226	0.9817	0.985022
129	0.8324366	0.887855	0.887756	0.906024	0.981681	0.983905
132	0.8318957	0.884093	0.883521	0.902186	0.981559	0.983681
135	0.8318576	0.882109	0.88006	0.898432	0.981543	0.982495
138	0.8308645	0.880252	0.876286	0.892353	0.981495	0.981454
141	0.8305763	0.875205	0.873129	0.892294	0.981383	0.981398
144	0.8303859	0.872158	0.869064	0.888712	0.981379	0.98124
147	0.8274875	0.867571	0.865737	0.885871	0.981238	0.980528
150	0.8269195	0.864925	0.863022	0.880345	0.981177	0.9801
153	0.8254998	0.86009	0.859673	0.875836	0.981043	0.979889
156	0.8253994	0.859037	0.856457	0.875702	0.980834	0.979254
159	0.8248107	0.858614	0.854145	0.871563	0.980799	0.97901
162	0.8235121	0.858127	0.85119	0.868011	0.980712	0.978796
165	0.8231831	0.855397	0.8489	0.865347	0.980613	0.978618
168	0.8214067	0.855312	0.845913	0.865144	0.9806	0.978041
171	0.8212647	0.855191	0.843154	0.862564	0.980568	0.977758
174	0.8207972	0.855042	0.840757	0.862179	0.980529	0.977725
177	0.8199627	0.850133	0.838297	0.858913	0.980417	0.977247
180	0.8191697	0.848313	0.835851	0.858746	0.980391	0.976533
183	0.8185948	0.847794	0.833248	0.856116	0.979945	0.976272
186	0.8079369	0.843123	0.830981	0.853452	0.979827	0.976203
189	0.7978157	0.842911	0.828468	0.853358	0.979737	0.974853
192	0.7968288	0.838033	0.826518	0.852312	0.97948	0.974559
195	0.7965795	0.833732	0.824112	0.852075	0.979073	0.974246
198	0.7962955	0.829515	0.821429	0.850329	0.978851	0.974009
201	0.7901752	0.827695	0.819645	0.850067	0.977398	0.973825
204	0.7898304	0.826991	0.817887	0.849845	0.9771	0.973555
207	0.7881176	0.826658	0.815325	0.849786	0.976926	0.973354

210	0.7867497	0.824833	0.813447	0.849727	0.97691	0.972428
213	0.7856659	0.823859	0.811399	0.849179	0.976551	0.971654
216	0.7847205	0.823552	0.809431	0.848977	0.976359	0.970277
219	0.7817812	0.822579	0.807736	0.848854	0.976096	0.969862
222	0.78092	0.822156	0.8058	0.848804	0.975387	0.969068
225	0.7807656	0.822119	0.804025	0.84879	0.975162	0.968913
228	0.7806378	0.821769	0.802343	0.848607	0.975117	0.968103
231	0.780065	0.821373	0.800836	0.848489	0.974899	0.967645
234	0.7799106	0.818103	0.798998	0.848484	0.974241	0.96748
237	0.7783385	0.815294	0.797058	0.848276	0.974001	0.967325
240	0.7765281	0.813161	0.795331	0.848158	0.97392	0.967279
243	0.7657066	0.809918	0.793601	0.847916	0.973872	0.966604
246	0.7568417	0.804405	0.792434	0.847906	0.97368	0.966472
249	0.7552627	0.79984	0.790783	0.847448	0.973506	0.966439
252	0.7544939	0.795787	0.789138	0.847216	0.973102	0.965306
255	0.753978	0.794771	0.78747	0.846111	0.973012	0.964891
258	0.753978	0.793168	0.785694	0.84581	0.972489	0.964255
261	0.7514431	0.792475	0.784612	0.845721	0.972409	0.963761
264	0.749168	0.791835	0.782783	0.845351	0.971906	0.963524
267	0.7489534	0.791189	0.781075	0.845129	0.971049	0.962964
270	0.7466921	0.789301	0.779649	0.844433	0.97103	0.962928
273	0.7452758	0.789195	0.778531	0.844034	0.970597	0.962216
276	0.7410788	0.788629	0.777055	0.843999	0.970289	0.961413
279	0.7331627	0.788613	0.775642	0.843555	0.970173	0.959739
282	0.732768	0.788317	0.774689	0.842993	0.970003	0.959472
285	0.7322797	0.788195	0.773298	0.842889	0.969839	0.958949
288	0.7318365	0.786486	0.77206	0.842771	0.968903	0.958925
291	0.7305725	0.786311	0.770741	0.842697	0.968765	0.958731
294	0.7282489	0.7861	0.769457	0.842499	0.968691	0.958659
297	0.7281416	0.78592	0.769028	0.842272	0.968354	0.958309
300	0.7275079	0.785475	0.768116	0.842134	0.968264	0.957894
303	0.7265729	0.785137	0.766752	0.841927	0.968258	0.957572
306	0.7265591	0.782306	0.765665	0.841813	0.968226	0.957147
309	0.7259011	0.781089	0.765088	0.841577	0.96786	0.957084
312	0.7258146	0.781021	0.763599	0.841103	0.967741	0.956942
315	0.7254752	0.779174	0.762651	0.840565	0.967446	0.956692
318	0.7239619	0.779058	0.761824	0.840388	0.967437	0.956564
321	0.7202186	0.778354	0.761256	0.840052	0.967177	0.956531
324	0.7195295	0.778307	0.759838	0.837802	0.967164	0.955915
327	0.7185564	0.778174	0.758854	0.837452	0.967045	0.955898
330	0.7168838	0.778074	0.758009	0.836658	0.966153	0.955641
333	0.7118142	0.77764	0.757584	0.835829	0.966031	0.955595
336	0.7092102	0.777582	0.756314	0.834477	0.965499	0.955341
339	0.7064295	0.777481	0.755867	0.833826	0.964995	0.955084
342	0.7063775	0.777116	0.754865	0.830969	0.964642	0.954399
345	0.7057923	0.776677	0.753837	0.830353	0.963959	0.954366
348	0.7045388	0.776312	0.752996	0.830224	0.963725	0.95292

351	0.7041405	0.775862	0.752312	0.830027	0.963612	0.952499
354	0.7035138	0.775841	0.751328	0.8298	0.962659	0.951827
357	0.7033233	0.775524	0.750827	0.829657	0.962582	0.951504
360	0.7030532	0.775153	0.750036	0.829588	0.962339	0.951201
363	0.7013633	0.774624	0.749454	0.828922	0.962223	0.951003
366	0.7000613	0.77455	0.748627	0.828907	0.962156	0.950891
369	0.6999332	0.774302	0.747357	0.828655	0.962056	0.950868
372	0.6990675	0.773915	0.747035	0.827215	0.96195	0.950588
375	0.6975438	0.773825	0.746212	0.826939	0.960879	0.950572
378	0.6970521	0.773751	0.745877	0.826795	0.960462	0.950509
381	0.6957985	0.773693	0.745331	0.826307	0.960398	0.950499
384	0.6957431	0.773085	0.744365	0.826134	0.960138	0.950206
387	0.6939736	0.772989	0.743967	0.825819	0.960074	0.950044
390	0.6920586	0.772915	0.743091	0.825725	0.959971	0.948575
393	0.6917712	0.77291	0.742572	0.825636	0.959868	0.947979
396	0.6913349	0.772894	0.741879	0.825602	0.959868	0.946391
399	0.6900363	0.772683	0.741186	0.825582	0.959451	0.946213
402	0.6892537	0.77264	0.740949	0.825384	0.959384	0.945265
405	0.6883672	0.772524	0.739853	0.825227	0.95906	0.944593
408	0.6874184	0.7719	0.739424	0.825009	0.958498	0.943743
411	0.6855034	0.771677	0.738556	0.824906	0.957898	0.94285
414	0.6817324	0.771376	0.738297	0.824797	0.9576	0.942781
417	0.6729991	0.771042	0.737921	0.823969	0.957449	0.94258
420	0.670485	0.770265	0.736987	0.82384	0.957257	0.942573
423	0.6614782	0.770074	0.736495	0.823668	0.956881	0.942214
426	0.6588602	0.768746	0.735793	0.823648	0.956262	0.941934
429	0.6571046	0.768482	0.735502	0.822977	0.955755	0.941539
432	0.6569764	0.768011	0.735216	0.822118	0.955473	0.94144
435	0.6563185	0.767762	0.734389	0.820633	0.955351	0.941299
438	0.6562042	0.767693	0.733982	0.819883	0.954985	0.940574
441	0.655477	0.767254	0.733548	0.819045	0.954738	0.940465
444	0.6550788	0.767143	0.732859	0.81904	0.954588	0.939978
447	0.6506879	0.766043	0.732506	0.818788	0.954575	0.93897
450	0.6498637	0.765598	0.732224	0.818685	0.954549	0.938475
453	0.6470969	0.764826	0.731862	0.818675	0.954469	0.937458
456	0.6468199	0.76354	0.731303	0.818561	0.954402	0.936743
459	0.6448461	0.762223	0.730901	0.818398	0.954347	0.936512
462	0.644479	0.761255	0.730427	0.818394	0.954138	0.936176
465	0.6443959	0.758276	0.730064	0.817979	0.954135	0.935731
468	0.6439146	0.757747	0.729523	0.817905	0.954039	0.935613
471	0.6435302	0.756647	0.729009	0.817811	0.953545	0.935537
474	0.6429311	0.756371	0.728553	0.817298	0.95308	0.935531
477	0.6425121	0.755911	0.728517	0.815833	0.952977	0.935405
480	0.6416083	0.755879	0.727994	0.814753	0.952942	0.935366
483	0.641307	0.755731	0.727529	0.814254	0.952765	0.93531
486	0.6409365	0.755186	0.727408	0.813682	0.952698	0.934865
489	0.6404067	0.755128	0.727082	0.813568	0.952461	0.93475

492	0.6403998	0.754652	0.727001	0.813415	0.952165	0.934733
495	0.6403617	0.754382	0.726286	0.812981	0.952027	0.934674
498	0.6371897	0.753848	0.726093	0.812769	0.951748	0.934621
501	0.6368642	0.753673	0.72583	0.812434	0.951668	0.934262
504	0.6341008	0.75252	0.725382	0.81198	0.951222	0.934111
507	0.6325806	0.751181	0.725324	0.81197	0.951193	0.934042
510	0.6305687	0.749816	0.725078	0.811763	0.949891	0.934022
513	0.629329	0.748927	0.724434	0.811634	0.949833	0.93392
516	0.6284945	0.748674	0.72392	0.811408	0.949628	0.933811
519	0.6283282	0.748398	0.723093	0.811102	0.949345	0.933643
522	0.6281828	0.747796	0.723039	0.811092	0.948733	0.933083
525	0.6270089	0.74779	0.722708	0.811087	0.94812	0.932872
528	0.6241313	0.747658	0.722605	0.810712	0.947831	0.93275
531	0.6215992	0.747631	0.721979	0.810643	0.947504	0.932124
534	0.6214122	0.746838	0.721979	0.8105	0.944495	0.931228
537	0.6205126	0.746806	0.721165	0.810436	0.943843	0.93106
540	0.6193622	0.746478	0.721031	0.810278	0.942704	0.929858
543	0.6189882	0.745944	0.720459	0.809686	0.94153	0.929127
546	0.6181453	0.745748	0.720441	0.809187	0.94076	0.929061
549	0.6167435	0.74533	0.720217	0.809138	0.94058	0.928995
552	0.6161825	0.74488	0.719806	0.808891	0.939028	0.928142
555	0.6157262	0.74424	0.719166	0.808803	0.93863	0.927974
558	0.6152476	0.743421	0.71888	0.808773	0.93811	0.927371
561	0.6132703	0.742986	0.718809	0.808753	0.937093	0.927081
564	0.6131248	0.742733	0.718683	0.808679	0.935374	0.927074
567	0.6131013	0.74159	0.717901	0.808255	0.933243	0.927055
570	0.6129379	0.741081	0.717879	0.808196	0.930754	0.926986
573	0.6118401	0.740071	0.717664	0.808181	0.93059	0.926729
576	0.611498	0.739659	0.717445	0.807762	0.930025	0.925895
579	0.6112757	0.739527	0.717422	0.807515	0.929583	0.924976
582	0.6111268	0.738463	0.717346	0.806992	0.928585	0.924614
585	0.6107355	0.734892	0.717337	0.80675	0.928508	0.924199
588	0.6104896	0.734299	0.71706	0.80673	0.928453	0.923922
591	0.6100568	0.734172	0.716971	0.806439	0.92665	0.92349
594	0.6100464	0.733395	0.716908	0.806281	0.92648	0.923319
597	0.6100083	0.731834	0.716863	0.806242	0.9264	0.923168

Figure 5 E

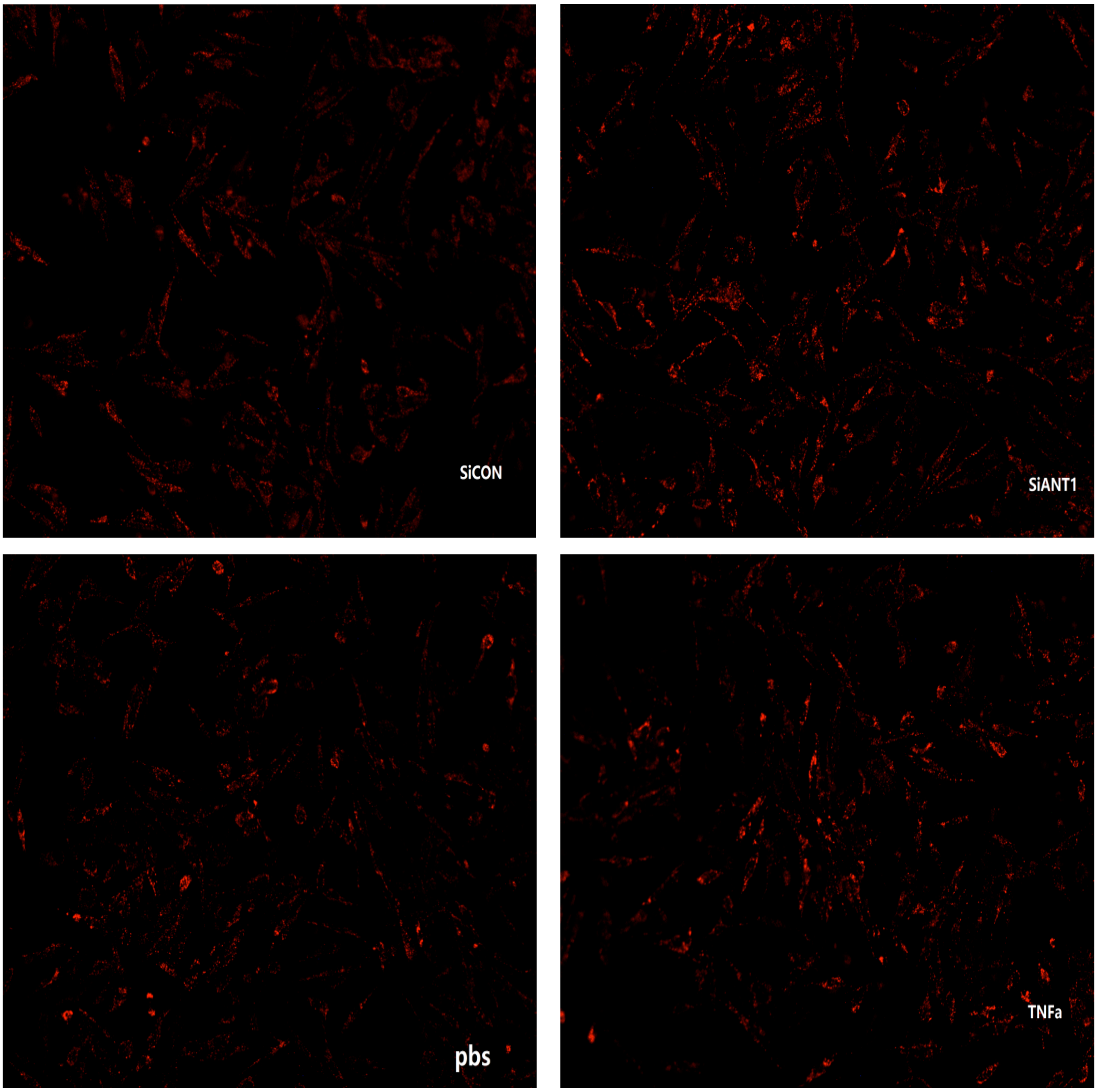

Figure 5 F

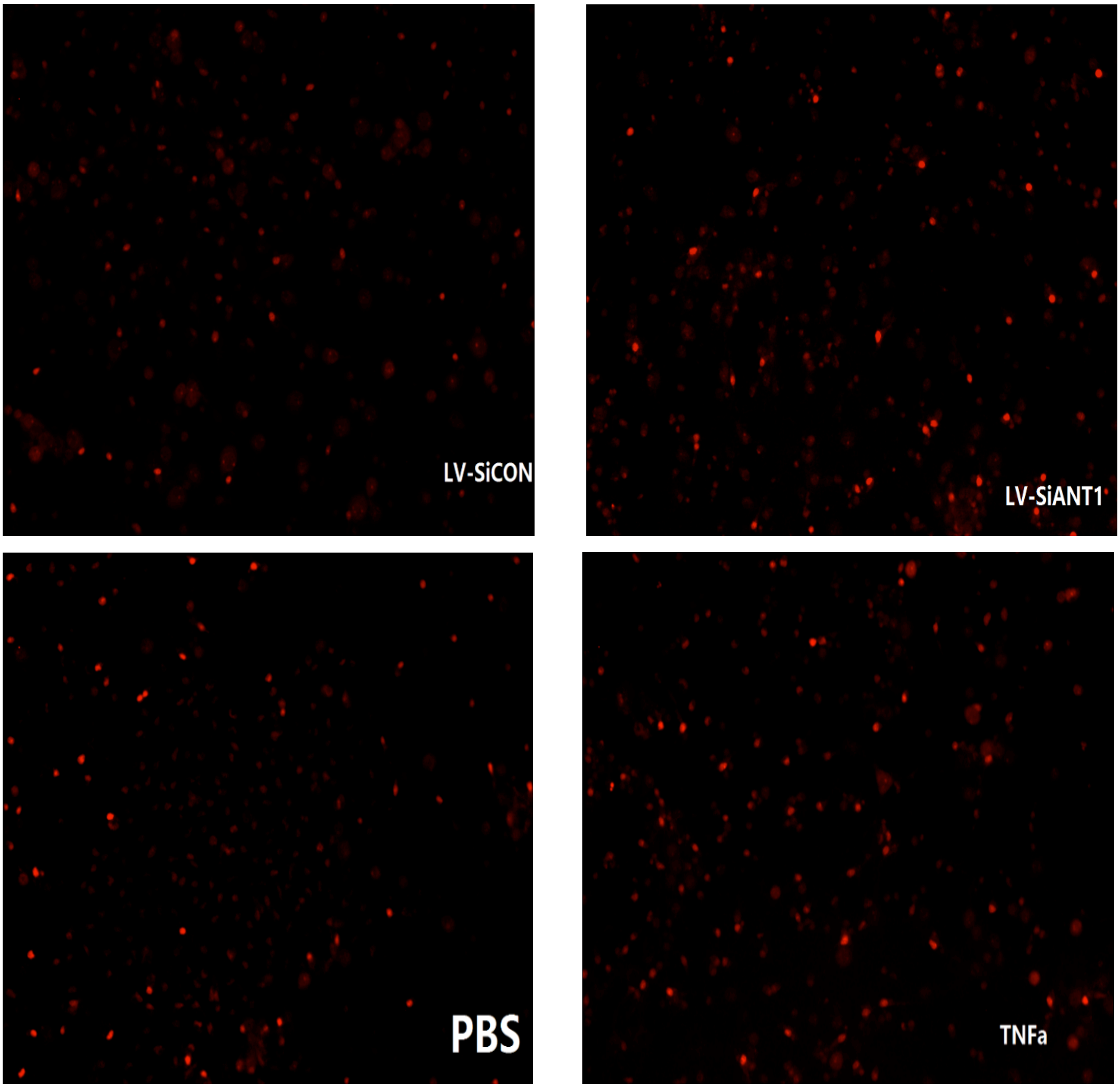

Figure 5 G

SiCON

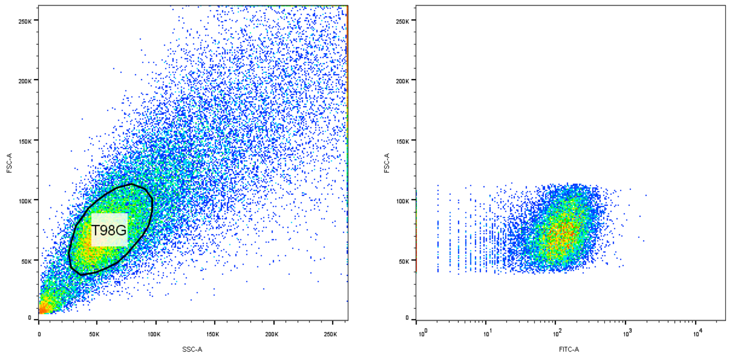

SiANT1

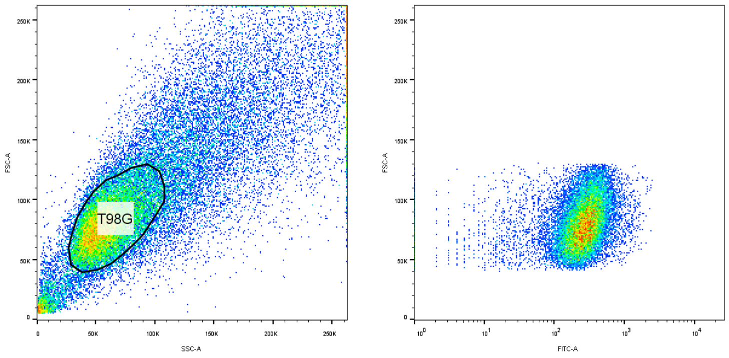

PBS

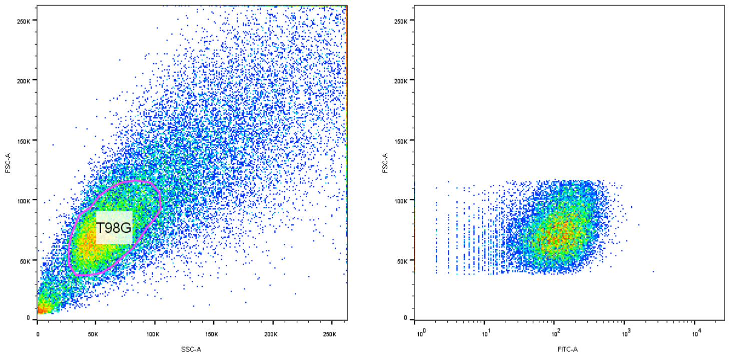

TNFa

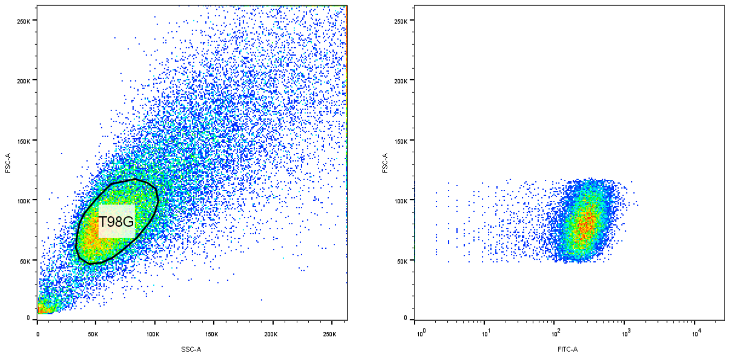

Figure 5 H

LV-SICON

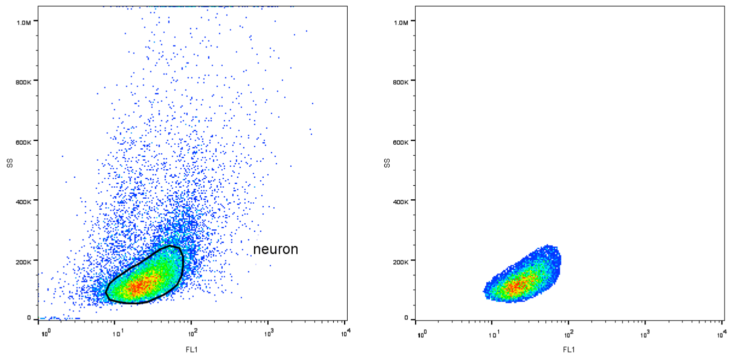

LV-SIANT1

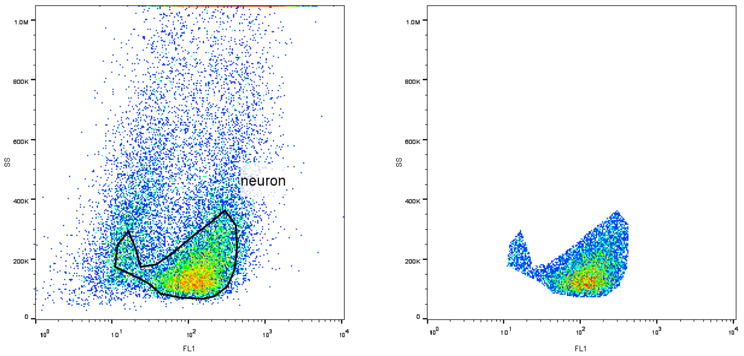

PBS

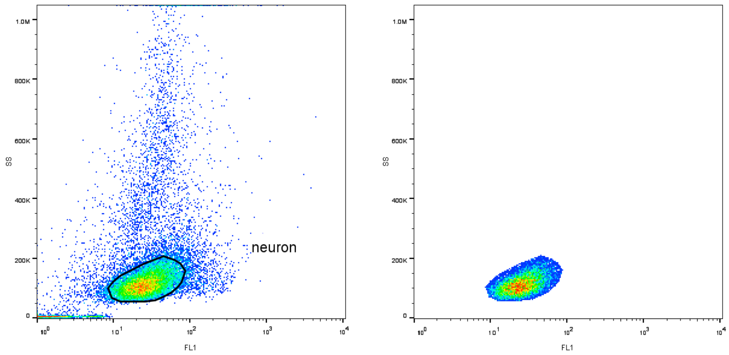

TNFa

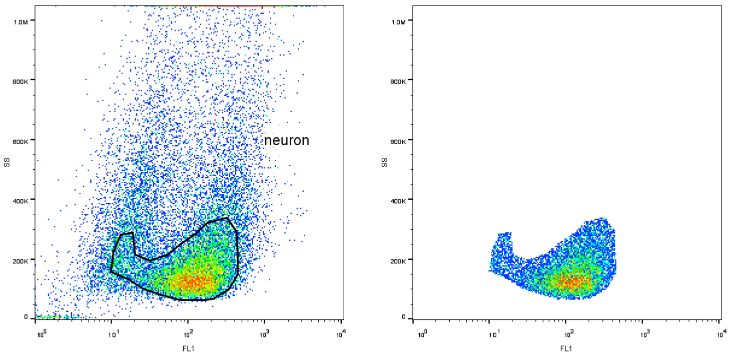

Figure 5 I

SiCON

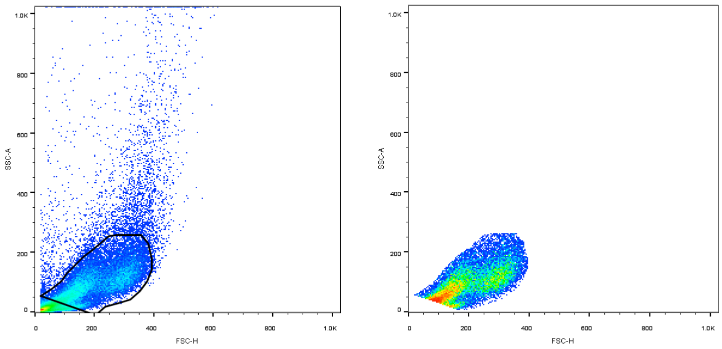

SiANT1

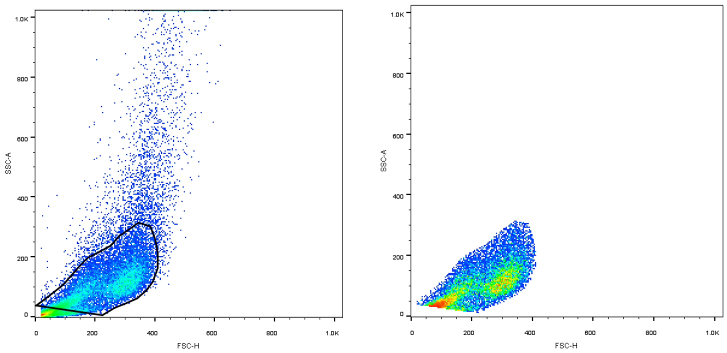

Figure 5 J

PBS

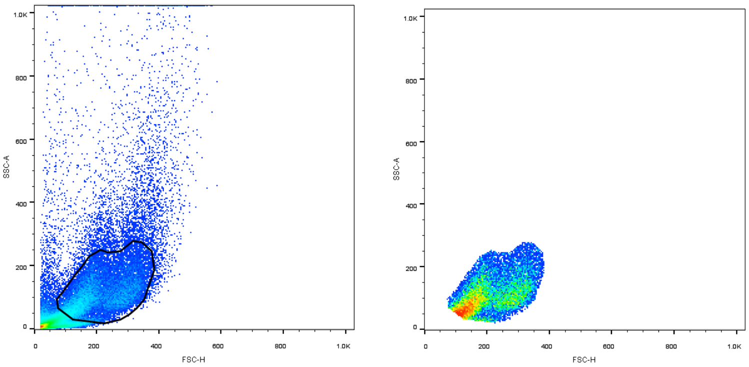

TNFa

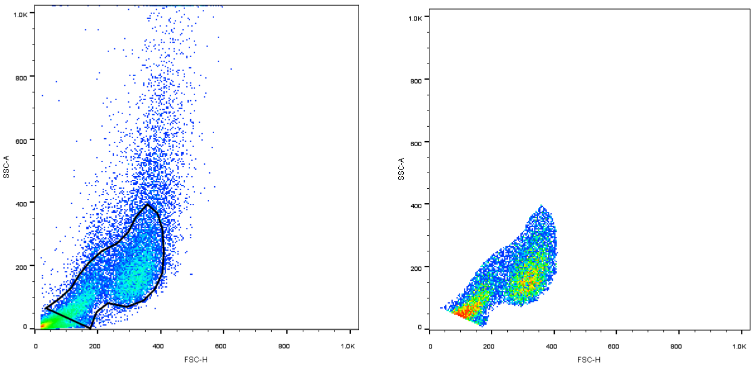

Supplement: Supplementary Information [file srep44708-s1.pdf]
